# The V-ATPase V_**0**_c' subunit is critical for *Candida albicans* pathogenicity in oral epithelial infection

**DOI:** 10.1080/20002297.2026.2739029

**Published:** 2026-09-30

**Authors:** Zheng Wang, Shenglan Long, Han Du, Xiaomeng Liu, Yiding Li, Qian Jiang, Lang Cai, Jing Zhou, Deqin Yang

**Affiliations:** a The Affiliated Stomatological Hospital of Chongqing Medical University, Chongqing Key Laboratory of Oral Diseases, Chongqing Technology Innovation Center of Smart Dental Medical Devices, Chongqing Municipal Key Laboratory of Oral Biomedical Engineering of Higher Education, Chongqing Municipal Health Commission Key Laboratory of Oral Biomedical Engineering, Innovation and Transformation of Dental Medical Devices Engineering Research Center of Chongqing Education Commission of China, Chongqing, China; b Department of Stomatology, Beijing Tongren Hospital, Capital Medical University, Beijing, China; c Department of Conservative Dentistry and Endodontics, Shanghai Stomatological Hospital and School of Stomatology, Fudan University, Shanghai, China

**Keywords:** *Candida albicans*, V-ATPase V_0_c' subunit, cAMP-PKA pathway, oral candidiasis, Bafilomycin A1

## Abstract

**Background:**

The vacuolar-type H^+^-ATPases (V-ATPase) are a multi-subunit enzyme located in the *Candida albicans* (*C. albicans*) vacuolar membrane. It is responsible for ion transportation and modulates several cellular responses, with the key structure being the c ring in the V_0_ domain. However, c ring’s role in oral epithelial infection remains unclear.

**Objective:**

This study aimed to reveal the role of V_0_c' subunit (encoding the gene *VMA11*), one of the subunits assemble c ring for oral Candidiasis *in vitro* and *in vivo*.

**Materials and methods:**

*VMA11* deletion and complementation mutants were constructed to evaluate the oral epithelial infection abilities of V_0_c' subunit, and western blotting and RT-qPCR were used to assess the regulate pathways for hyphal formation. Human oral mucosal keratinocyte (HOK) cells infected by *C. albicans* were subjected to transcriptome sequencing, and further validated using western blotting and immunofluorescence. Then, the impact of c ring specific inhibitor Bafilomycin A1 on oral epithelial infection by *C. albicans* were examined.

**Results:**

A *VMA11*-deleted mutant displayed reduced ATP contents, which decreased lower Ras and cyclic adenosine monophosphate (cAMP) levels, leading to inhibited hyphal growth by affecting the cAMP-protein kinase A (PKA) pathway. Additionally, the absence of *VMA11* further weakened *C. albicans* adhesion and damage capabilities toward oral epithelial cells, resulting in a diminished epithelial response associated with mitogen-activated protein kinase (MAPK) signaling. Bafilomycin A1 was then proved to effectively protect oral epithelium from *C. albicans* infection, which was consistent with the phenotypes of *VMA11* deletion mutant.

**Conclusion:**

Our findings highlight the crucial functions of V-ATPase V_0_c' subunit in oral epithelial infection and suggest that targeting the V-ATPase c ring may be a promising antifungal strategy against oral candidiasis.

## Introduction

According to recent evidence, the annual incidence of invasive fungal infections is 6.5 million individuals, with 3.8 million deaths [1], far exceeding the number of deaths caused by malaria and tuberculosis, and incurring a considerable medical burden on society. As a major conditional fungal pathogen, *Candida albicans* induces epithelial infections in immunocompromised populations with acquired immune deficiency syndrome (AIDS), who are receiving chemoradiotherapy and organ transplantation [2,3]. The oral cavity is one of the most common *C. albicans* colonization sites, where *C. albicans* detection rate is approximately 50% in healthy individuals [4,5]. During infection, *C. albicans* damages the mucosal barrier and induces oral candidiasis, particularly in the elderly, newborn, and denture wearers [6]. More seriously, oral candidiasis may lead to mucosal malignancy and eventually progress to oral cancer [7,8]. Therefore, identifying the pathogenic mechanisms underpinning *C. albicans* infection is critical for identifying new antifungal treatments.

During infection, *C. albicans* adheres and invades oral epithelial cells via a switch from budding yeast to hyphae [9]. Moreover, *C. albicans* secretes virulent proteins, such as secreted aspartyl proteases and candidalysin, that damage epithelial membranes [10–12]. *C. albicans* also produces adhesins that bind to receptors on epithelial cell surfaces and trigger host response signalling pathways [13]. Current clinical antifungal drugs include azoles, polyenes and echinocandins, which target ergosterol biosynthesis in cell membranes and 1,3-*β*-D-glucan synthase in cell wall biosynthesis, respectively [14,15]. However, the emergence of drug resistance and side effects has hindered antifungal treatments [16]. Thus, further studies are required to develop new antifungal targets and characterize their physiological functions.

Vacuolar-type H^+^-ATPases (V-ATPase) is a multisubunit enzyme present in the fungal vacuole and endomembrane system. V-ATPase is mainly involved in proton transport, pH regulation, organelle acidification, proteins delivery and autophagy [17]. There are two major V-ATPase domains: a cytoplasmic V_1_ domain consisting of eight subunits (A, B, C, D, E, F, G and H) and a membrane-bound V_0_ domain consisting of six subunits (a, c, c', c'' and d) [18,19]. The V-ATPase V_0_ domain provides the proton transport site, while the V_1_ domain is responsible for ATP hydrolysis and the energy supply for proton transport against the concentration gradients [20,21]. Thus, efficient V-ATPase subunit collaboration is critical for cellular homeostasis, and any subunit defects can impair *C. albicans* cellular processes. For example, subunit C mutation in the V_1_ domain causes growth inhibition, hyphal development defection and vacuolar dysfunction in the fungus [22]. Another study also reported that the c subunit of the V_0_ domain, encoded by *VMA3*, was necessary for aspartyl protease and lipase secretion, as well as vacuolar membrane fission [23]. Additionally, disrupted E and G abolished vacuolar functions and hyphal morphogenesis, leading to attenuated virulence in a mouse model [24].

In *C. albicans*, proton transport is mainly accomplished via the c ring consisting of c, c' and c'' subunits. The V_1_ domain hydrolyzes ATP, inducing c ring rotation, with protons in the cytoplasm entering the V_0_ domain through the cytoplasmic channel between the ‘a’ subunit and c ring. This sequentially neutralizes conserved glutamate residues on each subunit of c ring and enter the organelles through a membrane channel. Currently, c ring actions in oral diseases are poorly understood. We previously reported that the V_0_c' subunit of c ring, encoded by *VMA11*, was crucial for the synergistic cariogenic interaction between *C. albicans* and *Streptococcus mutans* [25]. However, it remains unclear how V_0_c' subunit affects *C. albicans* pathogenicity, particularly during oral epithelial infection. Importantly, no *C. albicans* V_0_c' subunit orthologs have been identified in mammals, suggesting that the V_0_c' subunit may be a potential antifungal therapy target [26].

In this study, we observed that the V_0_c' subunit was required for growth, vacuole morphogenesis and stress response in *C. albicans*. *VMA11* loss inhibited hyphal growth and influenced the signal transduction of cAMP-PKA pathway, leading to incapacitated oral mucosal infection *in vivo* and *in vitro*. Furthermore, we revealed that the specific c ring inhibitor bafilomycin A1 affected hyphal formation of *C. albicans* and protected mice from oral candidiasis.

## Materials and methods

### Strains and culture conditions

All *C. albicans* strains used in this study are listed in Table 1. A wild-type (WT) strain SN250 and *VMA11* deletion mutant *vma11Δ*/*Δ* were kindly provided by the Chongqing Key Laboratory of Oral Diseases (Chongqing Medical University, Chongqing, China). The complementation strain *vma11Δ*/*Δ*+*VMA11* was constructed using the pSFS2A plasmid, and the details are described in Supplementary data. The primers used for complementation strain construction are listed in Table 2.

**Table 1.** *Candida albicans* strains used in this study.

Strain	Parent	Genotype	Reference
SN250	CAI4	*ura3:imm434:URA3-IRO1/ura3:imm434 arg4:hisG/arg4:hisG his1:hisG/his1:hisG leu2:hisG:CdHIS1/leu2:hisG: CmLEU2*	Nobile and Mitchell [27]
*vma11Δ*/*Δ*	SN250	As SN250, but *vam11:LEU2/vma11:HIS1*	Jiang et al. [25]
*vma11Δ*/*Δ*+*VMA11*	*vma11Δ*/*Δ*	As *vma11Δ*/*Δ*, but VMA11p-VMA11	This study
CCC-32	Clinical strain	–	Liang et al. [28]
CCC-39	Clinical strain	–	Liang et al. [28]

**Table 2.** Primers used to generate a complementation strain.

Name	Sequence (5’ →3’)
*VMA11* 5'flank top + Apa I site	ATATACGGGCCCATACATCTCGCTTGCTTGTT
*VMA11* 5'flank bottom + Xho I site	ATATACCTCGAGGGAATTATCAGGTGATGCAT
*VMA11* 3'flank top + Sac II site	ATATACCCGCGGATGACGTGGTATGGATAGTT
*VMA11* 3'flank bottom + Sac I site	ATATACGAGCTCATTGATAAGGCATCCGGTAA

For routine growth, a single *C. albicans* colony was taken from a YPD plate (1% yeast extract, 2% peptone, 2% glucose and 2% agar), inoculated into liquid YPD medium, and grown overnight at 35 °C. *C. albicans* cells were harvested at the mid-logarithmic phase by centrifugation (6000 × g, 4 °C, 10 min), washed with phosphate buffered saline (PBS), counted using a hemacytometer (MARIENFELD, Germany) and finally resuspended in fresh YPD medium to the desired concentration.

### Hypha induction

*C. albicans* cells at the mid-logarithmic phase were inoculated into fresh spider medium (1% nutrient broth, 1% mannitol and 0.2% dipotassium phosphate) or YPD medium contained 10% serum (1 × 10^5^ cells/ml) and grown at 37 °C for 24 h in a shaking incubator (220 rpm). An inverted microscope (Leica, Germany) was used to visualize hyphae and record images. The colony morphology, effects of exogenous cAMP and bafilomycin A1 on hypha induction are described in Supplementary data. For each group, 5 fields were randomly selected to calculate the percentage of filamentous cells, and 30 fungal cells were randomly selected to count the hyphal length using imageJ software.

### Transmission electron microscope of thin sections to visualize *C. albicans* structure

*C. albicans* pre-cultured in YPD medium were harvested at the mid-logarithmic phase, fixed in 3% glutaraldehyde at room temperature, and incubated for 90 min in 1% osmium tetroxide. The samples were then serially dehydrated in acetone and embedded in epoxy resin (Epon812). Thin sections (approximately 50 nm) were prepared using an ultramicrotome and stained with 0.5% uranyl acetate and 0.5% lead citrate. The samples were observed in a JEM-1400PLUS TEM (JEOL, Ltd., Japan).

### Acidic, alkaline, metal ion and oxidative stress challenge

To investigate *C. albicans* tolerance to acidic, alkaline, metal ion and oxidative stress conditions, mid-exponential phase cells were gradient diluted in PBS, then 100 μl of the dilutions were plated onto YPD plates containing the following conditions: pH 5.0 (acidic), pH 7.0, pH 10.0 (alkaline), 100 μmol/L ZnCl_2_ (metal ion) and 5 mM hydrogen peroxide (H_2_O_2_) (oxidative stress) in triplicate for colony forming unit (CFU) counts [22].

### Reactive oxygen species (ROS) measurement of *C. albicans*

Mid-logarithmic phase *C. albicans* cells were harvested and resuspended (1 × 10^7^ cells/mL) in 5 mM H_2_O_2_ and incubated for 2 h. Then, *C. albicans* cells were washed three times with PBS to remove residual H_2_O_2_ and resuspended in dichlorodihydrofluorescein diacetate (H_2_DCF-DA) (10 µM, Beyotime, China) at 37 °C for 20  min. *C. albicans* cells were subsequently washed and resuspended (1 × 10^7^ cells/mL) in PBS. The fluorescence values (excited wavelength 488 nm/emitted wavelength 525 nm) were measured using a Thermo Scientific Multiskan GO reader (Thermo Fisher Scientific Inc., Waltham, MA, USA), and the fluorescence images were observed under fluorescence microscope (Leica, Germany).

### pH monitoring

Pre-cultured *C. albicans* cells were resuspended (1 × 10^5^ cells/mL) in fresh YPD medium. Then, 2 mL suspensions were transferred to a 24-well plate and incubated at 35 °C, after which pH values of *C. albicans* suspension were monitored at 1 h, 3 h, 6 h, 12 h, 24 h and 48 h (Corning pH meter 240; Corning Inc., NY).

### Assays of vacuolar morphology and chitin distribution

*C. albicans* cells of mid-exponential phase were collected and washed twice with PBS. The cell pellets were resuspended in PBS containing 2 µM FM4-64 (Beyotime, China) and labeled for 30 min with shaking. The cells were then harvested and washed three times with PBS. The pellets were finally resuspended in 500 ml of PBS, and 10 µl was examined by a fluorescence microscope (Zeiss, Germany). For each strain, cells in 6 randomly fields were selected to calculate the proportion of FM4-64 membrane-localized cells to total cells. For chitin distribution assay, *C. albicans* cells of mid-exponential phase were collected and stained with 50 μg/mL Calcofluor White (CFW, Beyotime, China) for 10 min. After being washed twice with PBS, *C. albicans* cells were observed via an inverted fluorescence microscope (TCS. SP8, Leica, Germany), and 30 cells from each group were randomly selected for quantitative analysis of cell wall fluorescence intensity using Image J software.

### RNA extraction and RT-qPCR detection of *C. albicans*

Mid-exponential phase *C. albicans* cells were resuspended in fresh YPD medium (1 × 10^5^ cells/mL) and cultured overnight. *C. albicans* cells were then harvested by centrifugation (4000 × g, 4 °C, 10 min), and total RNA was isolated using TRIzol Reagent (Invitrogen Life Technologies) according to the manufacturer’s instructions. RNA quality and integrity were determined using a NanoDrop spectrophotometer (Thermo Scientific, USA) and a Bioanalyzer 2100 system (Agilent, USA). RNA purification and reverse transcription were performed using a PrimeScript^TM^ RT Reagent Kit (Takara, Shiga, Japan). Real-time PCR was performed by a Bio-Rad CFX96 real-time PCR detection system with SYBR Green qPCR mix (TOYOBO, Japan). Data were analyzed based on the mean change in the qPCR cycle threshold compared to *ACT1* rRNA levels and reported using the 2^-ΔΔCT^ method. The primers used in this part are listed in Table 3.

**Table 3.** qPCR primers used for gene expression quantification in this study.

Primers	Sequence (5’ →3’)	Template strand
*ACT1* (Reference gene)	F R	TCCAGAAGCTTTGTTCAGAC TGCATACGTTCAGCAATACC
*RAS1*	F R	ATCAAGATGGATTAGCATTGG TGTTGTTGCTGTTGTTGTTG
*CYR1*	F R	AGAAAGAAGACGATGAAACAG AGGAGAACTAGAGGATGTAGAC
*TPK1*	F R	AGAACTTGCCAACAAACAAC TTTCTTGGTCAAGGAAAGAC
*TPK2*	F R	TTGTTGCCTGAACGTTCTAC CTACCATTGTGAACTGATCTC
*EFG1*	F R	ACAACCTCAGCATTACAATG ATAGGTACTGCTTGTTGACC
*FLO8*	F R	AGCAAATGACTAAGATGGCTG AGTCGGAATTACCAGTGTTTC
*CDC42*	F R	GATGGTGCCGTTGGTAAAAC AGCAGGAGAAATGACGGAAA
*CST20*	F R	ATGGGAAAAAGCGGAAAAGT TCCCACTCTATTGGCAAACC
*STE11*	F R	CGTGTAAATGCCACTGGTTG CTCCAACCGTTCTGGTGAAT
*HST7*	F R	TGCCCCTACATTGACAAACA ACACCTTCTTCGACCCCTTT
*CEK1*	F R	CATCATCAGCTTCAACAGCAA TGAAACTTGACGAGGGGAAG
*CPH1*	F R	ATGCAACACTATTTATACCTC CGGATATTGTTGATGATGATA
*TUB1*	F R	CCGGTTTGGGTTCTTTGTTA AAGTACAATCGGCGTGTTCC
*NRG1*	F R	CCCCATCCTTCCCAAGTACC TGGGTCTTTGCTTTGGGTGT
*RFG1*	F R	TAATGGAGGTGGAGGTGGAG TTGATGATGCATTGGTGGTT
*RBF1*	F R	CCCAGAGTCAACAACTGCAA CGTGCTCATTACCCTGTGTG
*ALS1*	F R	CCCAGAGTCAACAACTGCAA CGTGCTCATTACCCTGTGTG
*ALS3*	F R	CCCAGAGTCAACAACTGCAA CGTGCTCATTACCCTGTGTG
*SSA1*	F R	CCCAGAGTCAACAACTGCAA CGTGCTCATTACCCTGTGTG
*VMA11*	F R	ATCAGGTATTGGTATTGCTG CTAAATGCATGCACCCATTA
*GAPDH* (Reference gene)	F R	GGAGCGAGATCCCTCCAAAAT GGCTGTTGTCATACTTCTCATGG
*FOS*	F R	CCGGGGATAGCCTCTCTTACT CCAGGTCCGTGCAGAAGTC
*IL-6*	F R	ACTCACCTCTTCAGAACGAATTG CCATCTTTGGAAGGTTCAGGTTG

### Ras activity, PKA activity and intracellular ATP and cAMP content assays

For Ras activity assay, the mid-exponential phase *C. albicans* cells were resuspended in spider medium containing 0.2% glucose (1 × 10^5^ cells/mL) and cultured at 37 °C for 12 h. Then, *C. albicans* cells were washed with PBS and resuspended in radio immunoprecipitation assay (RIPA) lysis buffer (Beyotime, China) containing 1 mM protease inhibitor cocktail (Beyotime, China). Total protein was extracted using a FastPrep-24 homogenizer (MP Biomedicals, Santa Ana, CA, USA). Proteins were quantified by bicinchoninic acid (BCA) assay kit (Beyotime, China). All protein samples were then boiled for 10 min at 100 °C. Equal amounts of proteins (25 μg in total) were separated by 10% (w/v) sodium dodecyl sulfate‒polyacrylamide gel electrophoresis (SDS‒PAGE) (Epizyme, USA) and transferred to Immobilon®-FL transfer membranes (Millipore, USA). The membranes were blocked with 5% nonfat milk or 5% BSA for 2 h and incubated separately with 1:1000 diluted primary antibodies (Yeast *β*-actin, RRID: BE_4003; GTPase HRAS, RRID: AB_32417) at 4 °C overnight, respectively. After that, the membranes were incubated with HRP-tagged goat anti-rabbit IgG (1:3000, CST) for 2 h at room temperature. The membranes were then visualized using a BeyoECL Plus kit (Beyotime, China), followed by protein band quantification using Image J software.

The PKA activity, ATP and cAMP contents were detected following Assay Kit manufacturer’s instructions, and the details were described in Supplementary data.

### Cell lines

The human oral mucosal keratinocyte (HOK) cell line (kindly provided by Prof. Biao Ren from West China Hospital of Stomatology, Sichuan University) was routinely adherently cultured in high-glucose Dulbecco’s modified Eagle’s medium (DMEM, Thermo Scientific, USA) supplemented with 10% fetal bovine serum (FBS, Thermo Scientific, USA) and 1% penicillin–streptomycin antibiotics at 37 °C in a 5% CO_2_, humid environment.

### Epithelial cell adhesion and invasion assays

Epithelial cell adhesion and invasion assays were performed as described previously [4], and the details are described in Supplementary data.

### Epithelial cell damage assay

Lactate dehydrogenase (LDH) release was used to assess *C. albicans* damage toward HOK cells. HOK cells were inoculated into a 96-well plate with high glucose DMEM complete medium at 1 × 10^5^ cells/mL, and cultured for 24 h (37 °C, 5% CO_2_, humid environment). After removing the medium, cells were washed three times with PBS. *C. albicans* was then resuspended in high-glucose DMEM at 1 × 10^5^ cells/ml, and 100 μL of the suspension co-culture with HOK cells for 24 h at 37 °C in 5% CO_2_. The positive control group was treated with LDH release reagents at 24 h and cultured for a further 15 min. The negative control group was a ‘cells only’ group with no treatments. LDH levels were analyzed using an LDH Cytotoxicity Assay Kit with WST-8 (Beyotime, China).

### ROS measurement of epithelial cell

HOK cells were inoculated into a 96-well plate with high-glucose DMEM complete medium at 1 × 10^5^ cells/mL, and cultured for 24 h (37 °C, 5% CO_2_, humid environment). After removing the medium, cells were washed three times with PBS. Then, HOK cells were digested and resuspended in H2DCF-DA (10 µM, Beyotime, China) dissolved in serum-free DMEM medium, followed by incubation for 20 min in the dark (37 °C, 5% CO_2_, humid environment), and then washed three times with serum-free DMEM medium. Then, 100 μL of a *C. albicans* suspension resuspended in DMEM (1 × 10^5^ cells/mL) was added to the 96-well plate and co-cultured with HOK cells, while DMEM medium containing Rosup (5 mg/mL, Beyotime, China) was used as a positive control. The fluorescence values were measured every 15 min.

### Transcriptome sequencing

HOK cells were inoculated into a 6-well plate with high-glucose DMEM complete medium at 1 × 10^5^ cells/ml, cultured for 24  h (37 °C, 5% CO_2_, humid environment), and then co-cultured with *C. albicans* in high-glucose DMEM at 1 × 10^5^ cells/mL for 6 h. After removing the cell supernatant and washing three times with PBS, 1 ml of TRIzol lysis buffer was added for cell lysis, and the cells were collected and transferred to 1.5 mL cryovials. RNA extraction, library preparation and sequencing were performed by Shanghai Majorbio Bio-pharm Technology (Shanghai, China), and the details are described in Supplementary data. Sequence data files were deposited into the National Center for Biotechnology Information (NCBI) Sequence Read Archive (SRA) database (Accession Number: PRJNA1273333).

### RT-qPCR detection and western blot analysis of HOK cells

As described in the transcriptome sequencing protocol, HOK cells were co-cultured with *C. albicans* for 6 h. Then, RT-qPCR and western blot analysis of HOK cells were determined as described previously [29]. And the details were described in Supplementary data. The RT-qPCR primers used in this part are listed in Table 3. Primary antibodies (*β*-actin, RRID: AB_330288; c-FOS, RRID: AB_222699; IL-6, RRID: AB_6672) and HRP-tagged goat anti-rabbit IgG (1:3000, CST) were employed for western blot analysis.

### Murine oral candidiasis model

As previously described [28,30], the murine oral candidiasis model was used to study *C. albicans* pathogenicity *in vivo*. The protocol was approved by the Animal Ethics Committee of the Affiliated Stomatology Hospital of Chongqing Medical University (Ethics approval number: 2024-122, approval date: 25/09/2024). All experiments were performed in accordance with the National Institutes of Health Guidelines for the Care and Use of Laboratory Animals and Chongqing Medical University policies. Female BALB/c mice (4–6 weeks old and weighing approximately 20 g; 6 mice in each group) were sourced from Hunan SJA Laboratory Animal Co., Ltd. (Changsha, China, certificate: SCXK (XIANG)2024–0009). Briefly, the mice were subcutaneously injected with 4 mg of cortisone acetate (containing 5% Tween 80 dissolved in PBS) prior to and following infection. On the day of infection, the mice were intraperitoneally injected with 5% chloral hydrate (10 mL/kg). *C. albicans* (1 × 10^7^ cells/ml) were resuspended in sterile normal saline, which was soaked in cotton balls and placed under the tongue. The mice remained unconscious for at least 90 min. After 2 d, the mice were humanely euthanized, and the tongue infection area ratio was calculated using imageJ software, with the formula defined as the infected area divided by the total dorsal tongue area. The tongues were then longitudinally divided into two sections; one section was used for CFU counts, where prepared homogenates were plated onto CHROMagarTM *Candida* plates, while the other section was used for histopathology/immunohistochemical analysis. To examine bafilomycin A1 inhibitory activity against oral candidiasis, 50 μL bafilomycin A1 (0.5  μM) or equal DMSO volumes were coated onto tongues the day after infection. Bafilomycin A1 treatment was performed three times a day for two days in a row, and mice were euthanized and tongues were collected for *C. albicans* count and histopathology analysis as described above. Additionally, major organs, including the tongue, liver, spleen, and kidney, were stained using hematoxylin and eosin (HE) for bafilomycin A1 toxicity analysis.

### Antifungal susceptibility testing, RT-qPCR detection and cytotoxicity test

Antifungal susceptibility test was conducted in a sterile 96-well plate. An overnight *C. albicans* culture was resuspended in YPD medium (1 × 10^4^ CFU/mL). Then, 98  μL of the suspension and 2 μL of Bafilomycin A1 were added to the plate and two-fold serially diluted to determine the minimum inhibitory concentration (MIC). DMSO was used as a negative control, while fluconazole and econazole were positive controls. The plates were incubated at 37 °C for 24 h. The lowest drug concentration at which no fungal growth was observed by the naked eye under the experimental conditions was recorded as the MIC.

For RT-qPCR detection, *C. albicans* cells treated by Bafilomycin A1 or DMSO for 24  h were harvested by centrifugation (4000 × g, 4 °C, 10 min), and total RNA was isolated, purified and reverse transcribed according to the manufacturer’s instructions. Real-time PCR was performed by a Bio-Rad CFX96 real-time PCR detection system with SYBR Green qPCR mix (TOYOBO, Japan). Data were analyzed based on the mean change in the qPCR cycle threshold compared to *ACT1* rRNA levels and reported using the 2^-ΔΔCT^ method. The primers used in this part are listed in Table 3.

A Cell Counting Kit-8 (Beyotime, China) was used to test the cytotoxicity of Bafilomycin A1, and the details are described in Supplementary data.

### Statistical analysis

All the experiments were independently repeated at least three times. One-way ANOVA and Student’s t-test were used for *in vitro* studies, while non-parametric Mann–Whitney analysis was used for *in vivo* studies. Statistical analysis was performed with SPSS software, version 20.0 (SPSS Inc., Chicago, IL, USA). Significant differences were accepted at *p* < 0.05. Figures were generated using GraphPad Prism7 software (version 7.00 for Windows; GraphPad Prism, Inc., La Jolla, USA).

## Results

### *VMA11* is required for vacuole morphology, growth, aciduricity and stress tolerances of *C. albicans*

To characterize the potential role of *VMA11* gene in vacuolar morphology and biological functions, we observed *C. albicans* organelles by TEM. WT cells contained a single large central vacuole surrounded by several smaller vacuole-like fragments, while the *vma11Δ*/*Δ* strains accumulated vacuolar membrane folds on the vacuole interior that formed numerous ‘fragmented’ vacuoles, and these sub-compartments were also clustered (Figure 1A). Endocytosis results showed that FM4-64 was gathered at vacuolar membranes in most cells of WT and *vma11Δ*/*Δ*+*VMA11*, exhibiting typical red circular fluorescence (Figure 1B), with 96.1% and 92.7% of cells exhibiting FM4-64 membrane-localized pattern, respectively (Figure 1C). In contrast, in the *vma11Δ*/*Δ*, FM4-64 was internalized into the vacuole, and red fluorescence had dispersed across the entire vacuole, with only 20.9% of the cells having membrane-localized FM4-64 staining (Figure 1B and C). In addition, we also observed the distribution of chitin in *C. albicans* and found that in the WT, chitin was evenly distributed in the cell wall with relatively weak fluorescent signals. However, the deletion of *VMA11* enhanced the fluorescence signals in the cell walls and extended into the cytoplasm (Figure 1D and E), indicating an altered cell wall phenotype in the mutant. Together, these results suggest that the *VMA11* gene is required for vacuolar morphology, endocytosis and cell wall function regulation.

**Figure 1.**
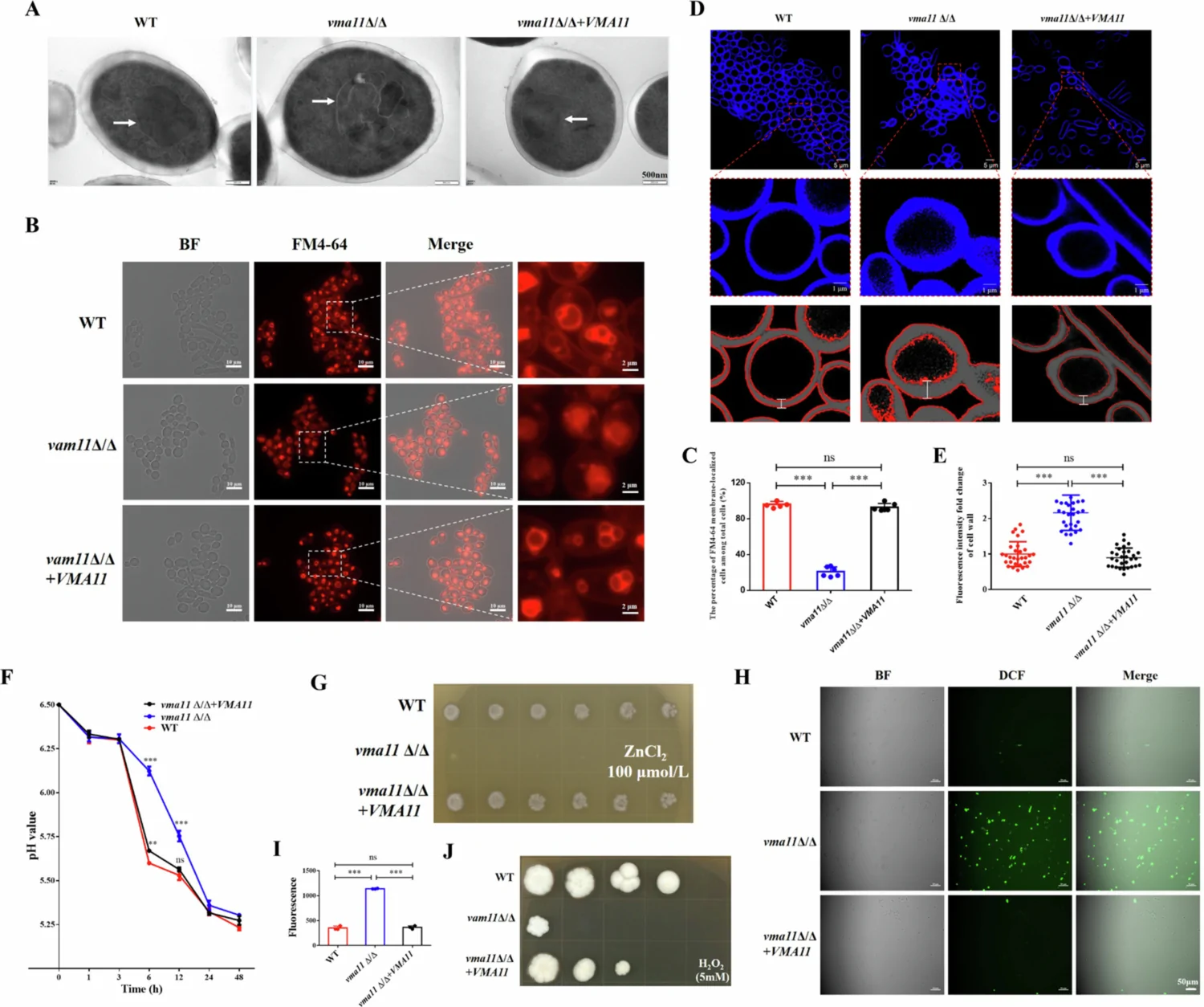
*VMA11* is required for vacuole morphology, aciduricity and stress tolerances of *C. albicans*. (A) Thin-section TEM images of *C. albicans* strains grown at the mid-logarithmic phase. (B) Endocytosis was assessed using FM4-64 staining, and the vacuolar membrane was stained red. (C) The percentage of FM4-64 membrane-localized cells among total cells in random fields (*n* = 6) (D). The distribution of chitin (stained blue) was observed by CLSM after CFW staining. (E) Fluorescence intensity fold change of the cell wall (*n* = 30). (F) The pH decreases of *C. albicans* strains over a period of 48 h (*n* = 3). (G) Growth of *C. albicans* strains under metal ion (ZnCl_2_) stress (*n* = 3). (H) ROS fluorescence images (stained green) of *C. albicans* strains treated with H_2_O_2_. (I) ROS intensity of *C. albicans* strains treated with H_2_O_2_ (*n* = 3). (J) Growth of *C. albicans* strains under oxidative stress (*n* = 3). (ns no significant, *** *p* < 0.001).

Consistent with our previous results [25], *vma11Δ*/*Δ* had a reduced growth rate with an extended logarithmic phase, and a decreased fungi yield at the stationary phase, while WT and *vma11Δ*/*Δ*+*VMA11* strains presented similar growth kinetics (Figure S1A). *vma11Δ*/*Δ* was also sensitive to both acidic and alkaline conditions (pH 5.0 and 10.0) when compared to WT (Figure S1B).

An aciduricity assay showed that although the pH levels of all the strains decreased to 5.25 at 48 h, the pH level of *vma11Δ*/*Δ* decreased significantly slower, with its pH remaining notably higher than that of the WT at both 6 and 12 h. Figure 1F, suggesting that *C. albicans* aciduricity was inhibited upon *VMA11* deletion. We next measured metal ion and oxidative tolerances in *C. albicans* strains. As expected, *vma11Δ*/*Δ* had a growth defect when exposed to ZnCl_2_, while these phenotypes were complemented in *vma11Δ*/*Δ*+*VMA11* (Figure 1G). An ROS release assay further indicated that elevated green fluorescence accumulation was observed in *vma11Δ*/*Δ*, which produced more reactive oxygen species (ROS) under H_2_O_2_ conditions (Figure 1H and I). Moreover, *vma11Δ*/*Δ* displayed a remarkable growth decrease after oxidants addition, while *vma11Δ*/*Δ*+*VMA11* cells grew to the level consistent with that of WT (Figure 1J). Taken together, these results imply critical roles for *VMA11* in vacuole morphology, growth, aciduricity and stress tolerances of *C. albicans*.

### The V_0_c' subunit regulates hyphal growth and affects signal transduction in the cAMP-PKA pathway

Hyphal formation is a core virulence attributes in the disease process where *C. albicans* infection occurs [31]. To assess V_0_c' subunit effects on filamentation, we performed a filamentation assay in liquid medium. In YPD medium supplemented with FBS, *vma11Δ*/*Δ* exhibited severely impaired hyphal length and filamentous cells proportion when compared with WT and *vma11Δ*/*Δ*+*VMA11*. In Spider medium, major *vma11Δ*/*Δ* cells remained in yeast form, while WT and *VMA11* complementation strain produced proper filaments (Figure 2A–E). Similar results were observed on solid plates, in which *vma11Δ*/*Δ* displayed smooth colonies and did not filament on both YPD and spider media. In contrast, the WT and *vma11Δ*/*Δ*+*VMA11* colonies appeared wrinkled and exhibited peripheral hyphal structures (Figure S2A). We next observed that *C. albicans* morphology by SEM and found that the *VMA11* deletion mutant failed to form typical hyphae comparable to those of the WT and complemented strain (Figure S2B). Together, these findings indicate that V_0_c' subunit activity is crucial for *C. albicans* filamentation.

**Figure 2.**
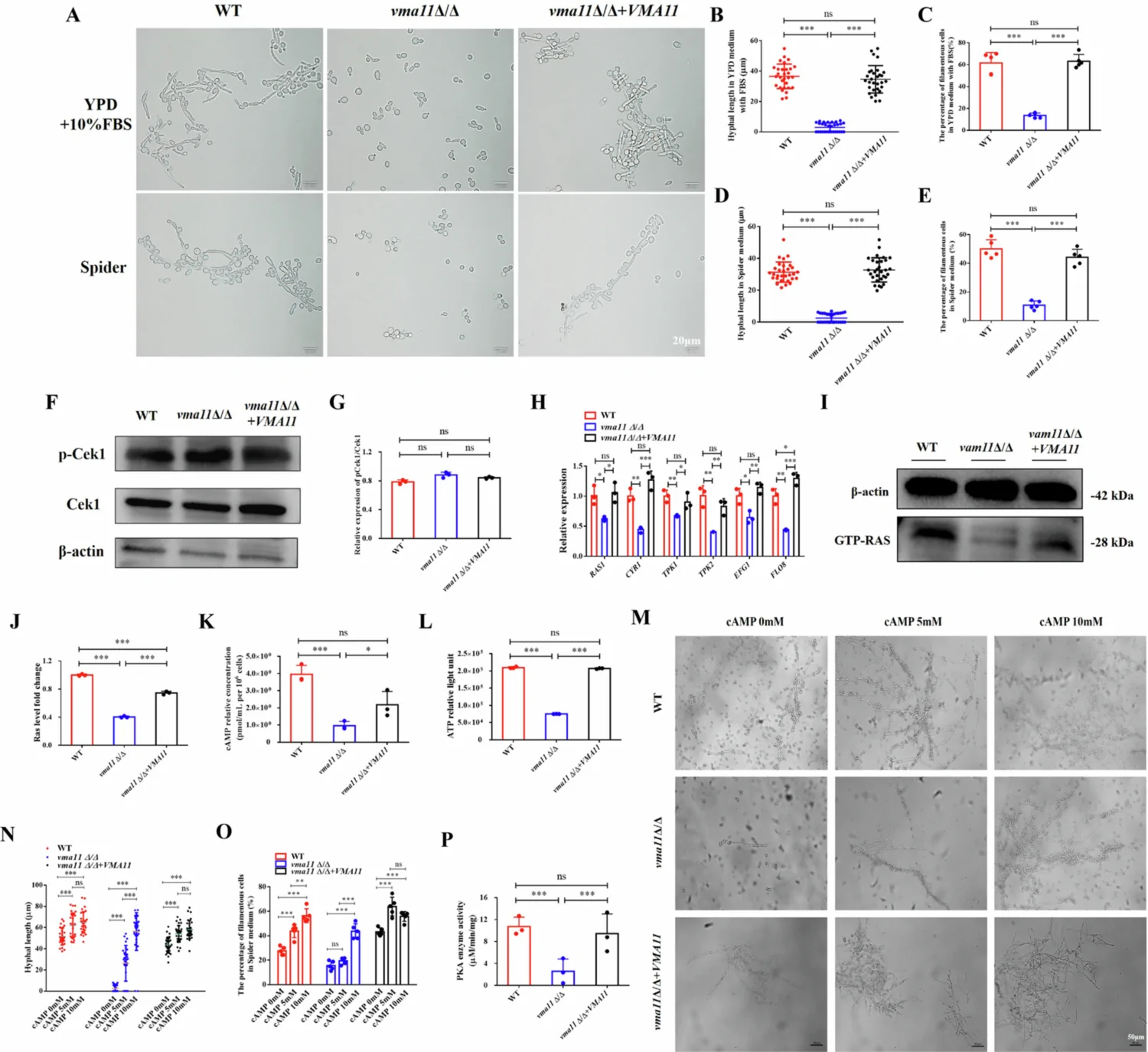
The V_0_c' subunit regulates hyphal growth and affects signal transduction in the cAMP-PKA pathway. (A) Hyphae formation of *C. albicans* strains in YPD and Spider liquid medium. (B) The hyphal length of *C. albicans* strains in YPD liquid medium (*n* = 30). (C) The filamentous cells percentage of *C. albicans* strains in YPD liquid medium (*n* = 5). (D) The hyphal length of *C. albicans* strains in Spider liquid medium (*n* = 30). (E) The filamentous cells percentage of *C. albicans* strains in Spider liquid medium (*n* = 5). (F) Representative western blotting images of *p*-Cek1 and Cek1 in *C. albicans* strains. (G) Quantification of *p*-Cek1/Cek1 in *C. albicans* strains (*n* = 3). (H) The relative expression of genes in cAMP-PKA pathway (*n* = 3). (I) Representative western blotting images of Ras in *C. albicans* strains. (J) Quantification of Ras in *C. albicans* strains (*n* = 3). (K) The cAMP relative concentration of *C. albicans* strains (*n* = 3). (L) The ATP production of *C. albicans* strains (*n* = 3). (M) Hyphae formation of *C. albicans* strains with the exogenous addition of cAMP. (N) The hyphal length of *C. albicans* strains treated by cAMP (*n* = 30). (O) The filamentous cells percentage of *C. albicans* strains treated by cAMP (*n* = 5). (P) The PKA activity of *C. albicans* strains (*n* = 3). (ns no significant, * *p* < 0.05, ** *p* < 0.01, *** *p* < 0.001).

*C. albicans* hyphal development is regulated by complicated regulatory networks. It is generally accepted that there are two major positive signalling pathways, cAMP-PKA pathway and the MAPK pathway, which are carried out by the transcription factors Efg1 and Cph1, respectively, and one negative regulation pathway carried out by a complex consisting of Tup1, Nrg1 and Rfg1 [32]. To better understand the relationship between V_0_c' subunit and *C. albicans* filamentation, we analyzed the alteration of Tup1 mediated negative regulation pathway. As shown in Figure S2C, *VMA11* deletion did not upregulate the expression of this negative regulation pathway, and Tup1 (general transcriptional corepressor) was significantly downregulated, while Nrg1 (DNA-binding protein) and Rfg1 (regulator of filamentous growth) displayed no significant differences between *vma11Δ*/*Δ* and WT. We then measured the positive regulation of MAPK pathway variation. Although the transcriptional levels of partial upstream proteins Ste11, Hst7 and Cek1 pronouncedly decreased in *vma11Δ*/*Δ* (Figure S2D), the phosphorylation level of Cek1 in *vma11Δ*/*Δ* showed no significant difference compared to WT (Figure 2F and G). In addition, previous studies have reported that the transcription factor Cph1 and its upstream proteins only participate in hyphal formation on solid Spider medium instead of liquid media [33,34]. Since *CPH1*, *CDC42* and *CST20* expression in the *vma11Δ*/*Δ* strain were not significantly different to WT, and *VMA11* disruption failed to drive filamentation in both solid and liquid culture media in our study, we speculated that the MAPK pathway may not be involved in regulating *VMA11* mediated hyphal formation.

We further examined whether *VMA11* functions in another positive regulation cAMP-PKA pathway. As expected, *VMA11* deletion significantly downregulated Efg1 (major regulator) transcriptional levels in this pathway. Additionally, upstream proteins (Cyr1, Tpk1, and Tpk2) were also inhibited in *vma11Δ*/*Δ* (Figure 2H). Signal transduction to the downstream transcription factors Efg1 is based on cAMP, which is activated by the upstream protein Ras1 and subsequently induces PKA phosphorylation to regulate the downstream multiple signals. Thus, we first quantified Ras level and found that the deletion of *VMA11* reduced Ras protein (Figure 2I and J). cAMP and its precursor ATP intracellular investigation further showed that *vma11Δ*/*Δ* had decreased cAMP and ATP levels when compared to WT (Figure 2K and L). As expected, stimulation with exogenous cAMP compensated for hyphal growth in *vma11Δ*/*Δ* (Figure 2M–O). Furthermore, we observed decreased PKA activity in *vma11Δ*/*Δ* (Figure 2P), suggesting that *VMA11* deletion-mediated filamentation involves extensive changes in cAMP-PKA pathway, including reduced Ras levels, decreased intracellular ATP and cAMP concentrations, and downregulation of PKA activity. Together, these results further support our assumption that *VMA11* is critical for *C. albicans* hyphal growth and induces significant variation in cAMP-PKA pathway.

#### The*vma11Δ*/*Δ*mutant exhibits defects in adhesion, invasion, and damage to oral epithelial cells, a process may involve the MAPK signaling pathway

During oral candidiasis development, *C. albicans* invades tissue through the adherence to epithelial cell surfaces and puncturing cells by hyphae [4]. *C. albicans* also secrete many virulence factors that destroy cell junctions and integrity, such as agglutinin-like sequence (Als) [35], invasin (Ssa1) [36] and fungal cytolytic peptide toxin (Candidalysin) [10]. To investigate whether *VMA11* mediated phenotypic variation affects the virulence toward oral epithelial cells, we examined cell adhesion ability in *C. albicans* strains. As shown in Figure 3A and B, *VMA11* deletion significantly impaired adhesion to oral epithelial cells when compared to WT and *vma11Δ*/*Δ*+*VMA11*. A similar tendency was also observed in the cell invasion assay where WT and complementary strain aggressively invaded and penetrated epithelial cells. In contrast, the loss of *VMA11* failed to invade or penetrate cells, and no visible fungal elements were detected in the cells (Figure 3C). RT-qPCR results further indicated that *VMA11* deletion significantly downregulated genes required for adhesion and invasion into human cells, including *ALS1*, *ALS3* and *SSA1* (Figure 3D). Such virulence alterations caused by *VMA11* deletion triggered a cascade of reactions, since the epithelial cell damage induced by *vma11Δ*/*Δ* was significantly reduced compared to WT and *vma11Δ*/*Δ*+*VMA11*(Figure 3E). And *vma11Δ*/*Δ*exhibited impaired ability to stimulate epithelial cell ROS production during the entire co-incubation process (Figure 3F), suggesting that *VMA11* plays an important role in epithelial infection.

**Figure 3.**
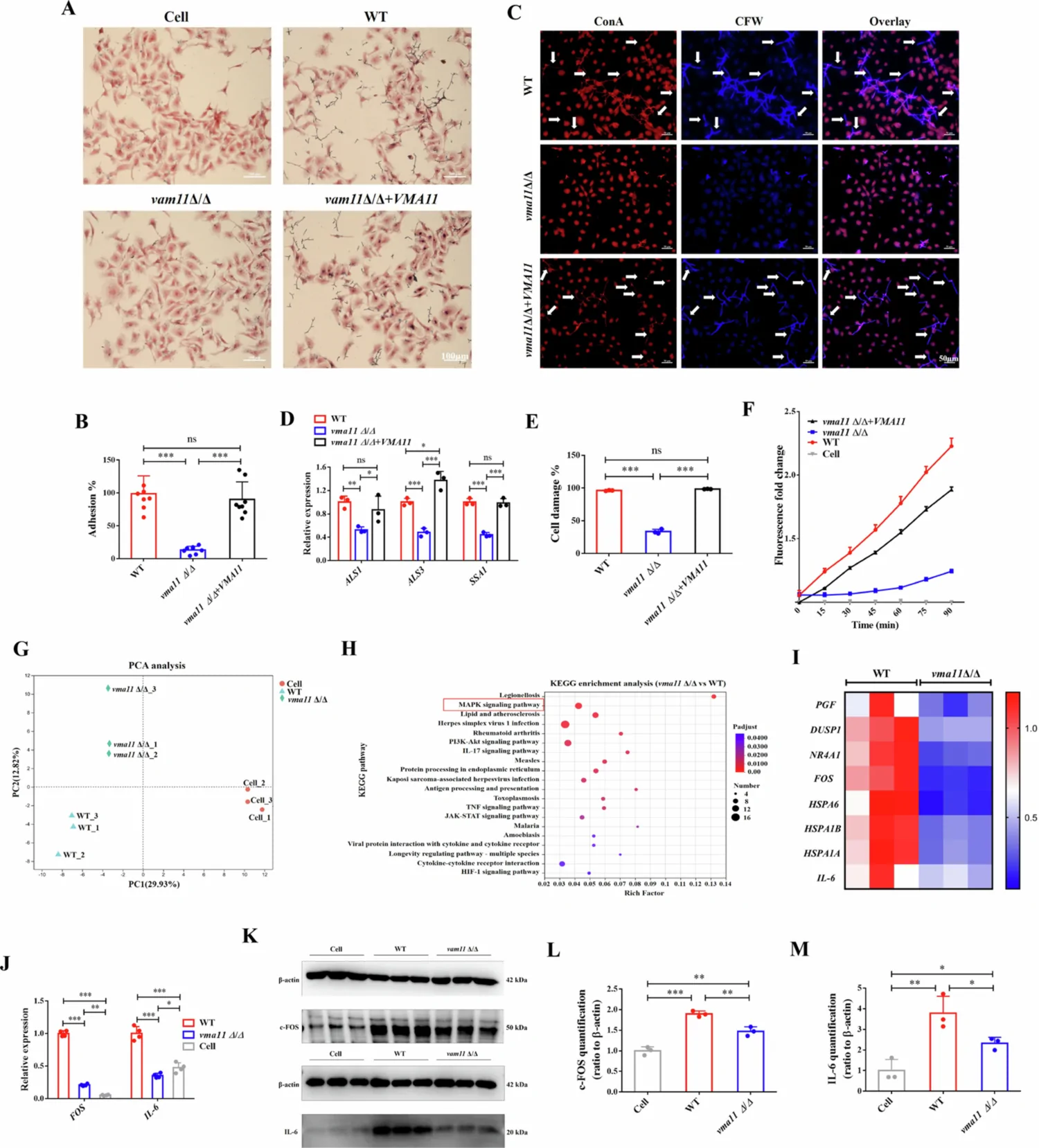
The *vma11Δ*/*Δ* mutant is defective in adhesion and invasion to oral epithelial cells, which may be involved in the MAPK pathway. (A) The images of *C. albicans* strains adhere to HOK cells. (B) The adhesion percent of *C. albicans* strains on HOK cells (*n* = 3). (C) The images of fluorescence staining of *C. albicans* hyphae invade into HOK cells. Cell wall chitin is stained with Calcofluor White (CFW, post-permeabilization), whereas carbohydrates are shown by Alexa-Fluor-647-labeled concanavalin A (ConA, pre-permeabilization), which respectively distinguish between invading hyphae and non-invading hyphae, and white arrows represent invading hyphae into HOK cells. (D) The relative expression of adhesion and invasion genes in *C. albicans* strains (*n* = 3). (E) HOK cell damage reflected by LDH release after 24  h post infection by *C. albicans* strains (*n* = 3). (F) ROS intensity of HOK cells treated by *C. albicans* strains over a period of 90  min (*n* = 3). (G) PCA analysis of HOK cells transcriptome treated by *C. albicans* WT and *vma11Δ*/*Δ*. (H) KEGG enrichment analysis between WT and *vma11Δ*/*Δ*. (I) Transcriptome heat map of MAPK pathway. (J) The relative expression of *FOS* and *IL-6* genes in HOK cells treated by *C. albicans* WT and *vma11Δ*/*Δ* (*n* = 3). (K) Representative western blotting images of c-FOS and IL-6 in HOK cells (*n* = 3). (L) The quantification of c-FOS in HOK cells (*n* = 3). (M) The quantification of IL-6 in HOK cells (*n* = 3). (ns no significant, * *p* < 0.05, ** *p* < 0.01, *** *p* < 0.001).

To better understand the mechanisms by which oral epithelial cells respond to *VMA11* deletion, we performed transcriptome sequencing of HOK cells infected by *C. albicans* strains. Significant differences were identified among each group according to principal component analysis (PCA) (Figure 3G), indicating that *C. albicans* infection affects the transcriptome of oral epithelial cells. Kyoto Encyclopedia of Genes and Genomes (KEGG) enrichment analysis showed that both WT and *vma11Δ*/*Δ* infection activated the MAPK, HIF-1, IL-17 and TNF signaling pathways in epithelial cells (Figure S3A and B). Among them, the differential genes mainly enriched in MAPK signaling pathway between WT and *vma11Δ*/*Δ* groups (Figure 3H and Table S1 in supplementary data). Additionally, the heat map indicated that the key genes of MAPK signaling pathway were downregulated after treated by *vma11Δ*/*Δ* (Figure 3I), which implied *vma11Δ*/*Δ* infection may influence the canonical MAPK signaling pathway. These variations were verified by further RT-qPCR and Western blotting, which showed that *vma11Δ*/*Δ* reduced the mRNA and protein levels of c-Fos in the MAPK pathway and the subsequent inflammatory factor IL-6 (Figure 3J‒M). These results imply that *VMA11* induces oral mucosal damage via the MAPK signaling pathway *in vitro*. These results indicate that *VMA11* can induce oral mucosal damage *in vitro* and that the MAPK signaling pathway may be involved in the host's immune response to this mucosal infection.

### *VMA11* loss strongly impairs *C. albicans* oral mucosal pathogenicity *in vivo*

To examine the potential *VMA11* roles in oral mucosal pathogenicity, we constructed mouse oropharyngeal candidiasis models using *C. albicans* strains. The epithelial infection area ratios in the mice inoculated with WT and *vma11Δ*/*Δ*+*VMA11*were 23.28% and 21.86%, respectively, while in the mice infected with the *vma11Δ*/*Δ* strain, the ratio was 0 (Figure 4A). As shown in Figure 4B, WT and *vma11Δ*/*Δ*+*VMA11* infection led to the formation of scattered white patterns (white patches) on tongue surfaces. In contrast, the *VMA11* disruption mutant failed to form white patches area, with almost no visible damage on tongue mucosa. Colonized *C. albicans* counts also indicated that *vma11Δ*/*Δ* displayed a significantly decreased fungal burden in the epithelial samples (Figure 4C).

**Figure 4.**
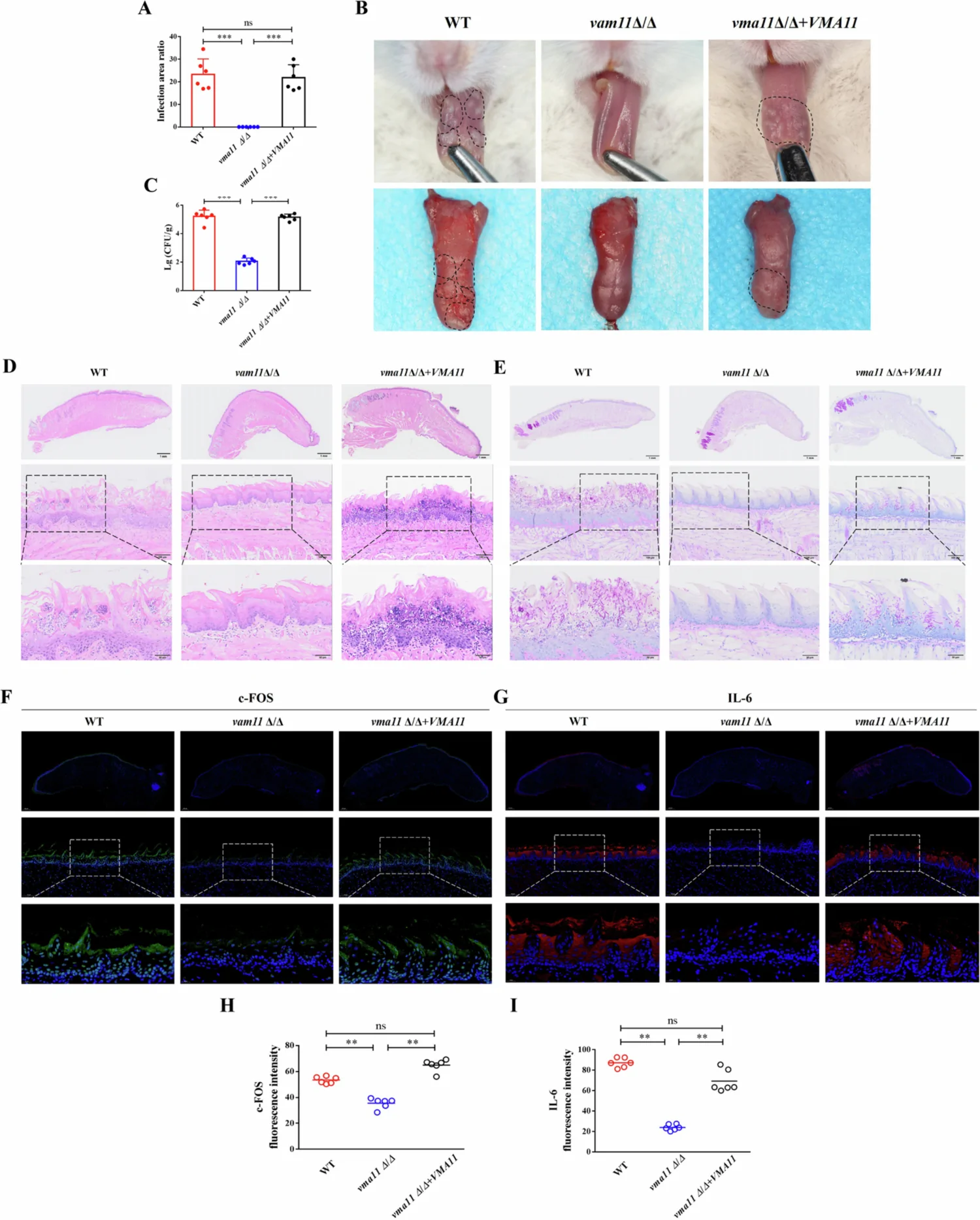
Loss of *VMA11* strongly impairs the oral mucosal pathogenicity of *C. albicans in vivo*. (A) The infection area ratio of the mice entire tongue (*n* = 6). (B) The images of infected mice tongues, leukoplakia on the tongue are labelled by dotted lines. (C) *C. albicans* burdens obtained from the tongues of mice after oropharyngeal infection (*n* = 6). (D) HE staining of tongue tissues of mice, inflammatory cells and infiltrating immune cells are showed in black box. (E) PAS staining of tongue tissues of mice, invading hyphae are showed in the black box. (F) c-FOS immunofluorescence staining of tongue tissues of mice. c-FOS, green; nucleus, blue. (G) IL-6 immunofluorescence staining of tongue tissues of mice. IL-6, red; nucleus, blue. (H) Fluorescence intensity of c-FOS from the tongues of mice (*n* = 6). (I) Fluorescence intensity of IL-6 from the tongues of mice (*n* = 6). (ns no significant, ** *p* < 0.01, *** *p* < 0.001).

From HE staining results, successive epithelial cell layers, without inflammatory cell infiltration, were observed in the *vma11Δ*/*Δ* group. However, WT and complementary strain caused corneal layer thickening, accompanied by mucosal epithelial cell exfoliation. Additionally, lamina propria integrity was destructed in the WT and *vma11Δ*/*Δ*+*VMA11* groups, with considerable neutrophilic lymphocytes and macrophages identified in infection areas (Figure 4D). Periodic Acid-Schiff (PAS) staining also confirmed the critical role for *VMA11* in epithelial infection *in vivo*. Multiple intraepithelial micro-abscesses consist of hyphae, necrotic cells and macrophage appeared in the hyphal invasion region in mice infected with WT and *vma11Δ*/*Δ*+*VMA11*. In contrast, no obvious invasive *C. albicans* hyphae or yeast forms were observed with *vma11Δ*/*Δ* (Figure 4E). The immunohistochemistry results were consistent with the *in vitro* findings and showed that c-FOS+cell numbers in tongue tissue were larger in the WT infection group when compared to the *vma11Δ*/*Δ* infection group. In addition, IL-6 also exhibited high expression levels in the corneal layer and lamina propria in the WT group but was not observed in the *vma11Δ*/*Δ* infected mice (Figure 4F–I). Taken together, these results indicated that *VMA11* is crucial for oropharyngeal candidiasis *in vivo*.

#### Targeting V-ATPase c ring inhibits *C. albicans* hyphal development and protects oral epithelial cells from fungal infection

Having identified crucial V_0_c' subunit roles in regulating *C. albicans* virulence, we further used bafilomycin A1 (Figure 5A), a small molecule that targets the V-ATPase c ring [37,38], to examine its inhibitory activity against oral candidiasis. As shown in Table 4 and Figue S4A, bafilomycin A1 significantly suppressed *C. albicans* growth (MIC of 0.5 μM) and downregulated *VMA11* expression level. Furthermore, bafilomycin A1 inhibited WT filamentation and the hyphal formation of clinical strains CCC-32 and CCC-39 (Figure 5B–D), which exhibited resistance and sensitivity to fluconazole, respectively (Figure S4B–D). As expected, almost all tested genes in the cAMP-PKA pathway were downregulated by bafilomycin A1 treatment (Figure 5E), indicating that bafilomycin A1 potentially repressed *C. albicans* hyphal formation via cAMP-PKA pathway. The effect of bafilomycin A1 on *C. albicans* virulence to HOK cells were further investgated, and *C. albicans* failed to adhere and invade into oral epithelial cells after bafilomycin A1 treatment (Figure 5F–H). Moreover, bafilomycin A1 was non-toxic to HOK cells at the MIC (0.5 μM, Figure S4E). The histological results from the mice were consistent with the *in vitro* phenotypes, which suggested that bafilomycin A1 significantly repressed *C. albicans* colonization and white patches formation in mouse tongues after infection (Figure 5I and J). HE and PAS stains showed substantially reduced epithelial inflammation and hyphae invasion upon bafilomycin A1 treatment (Figure 5K and L). In addition, bafilomycin A1 toxicity analyses indicated that its topical administration caused no damage to major organs, including the tongue, liver, kidney and spleen (Figure S4F). Thus, using bafilomycin A1 as a pharmacological model tool that interference with V-ATPase activity can inhibit *C. albicans* hyphal formation and its pathogenicity in oral epithelial infection both *in vitro* and *in vivo*.

**Table 4.** The inhibitory effects of bafilomycin A1 and fluconazole/econazole against *C. albicans* wild type.

Compounds	MIC
Bafilomycin A1	0.5 μM
Fluconazole	1.25 μg/mL
Econazole	1.25 μg/mL

**Figure 5.**
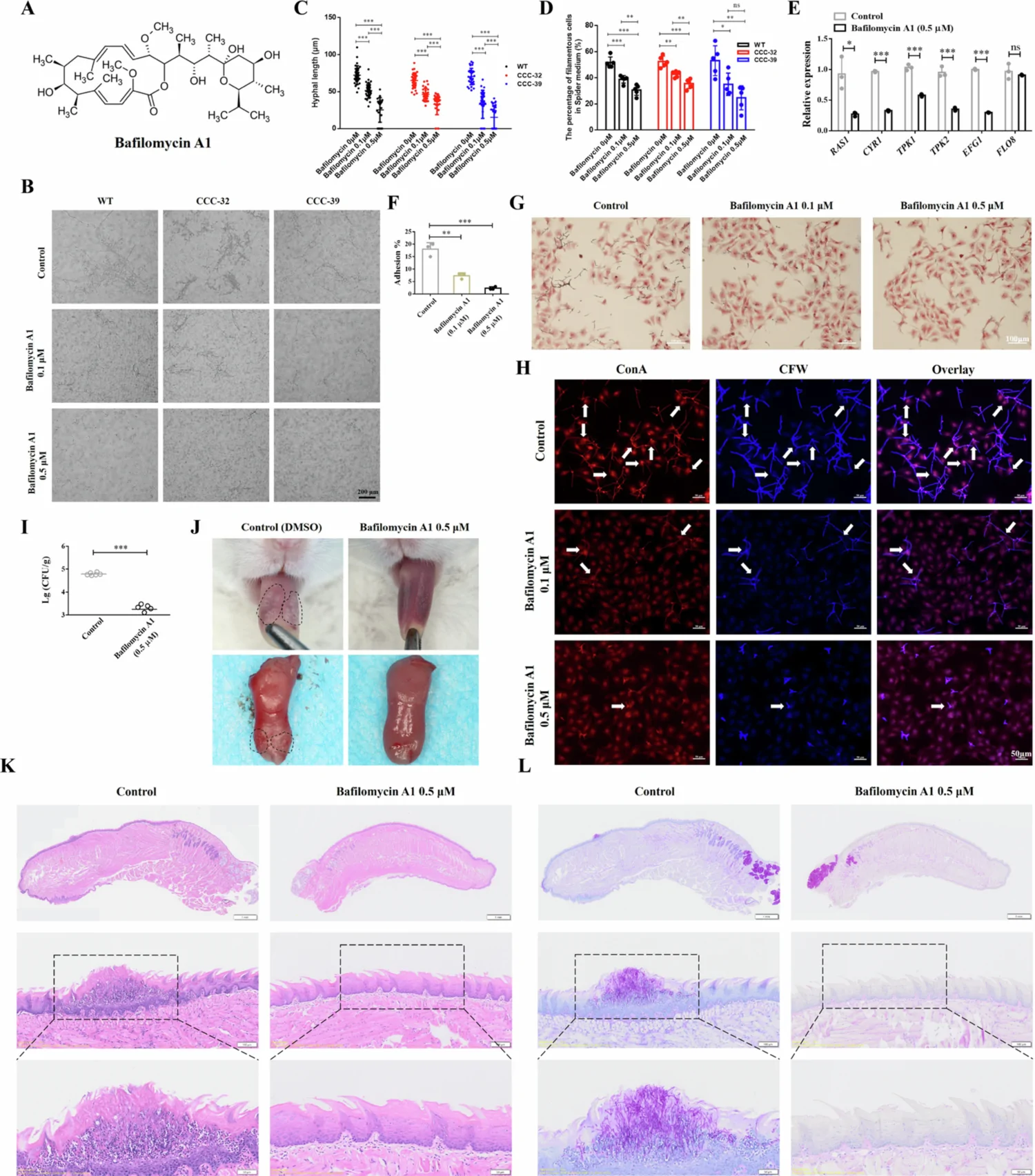
Targeting V-ATPase c ring inhibits *C. albicans* hyphal development and protects oral epithelial cells from fungal infection. (A) The chemical structure of compound bafilomycin A1. (B) Hyphae formation of *C. albicans* WT, CCC-32 and CCC-39 with the treatment of bafilomycin A1 in YPD medium. (C) The hyphal length of *C. albicans* WT, CCC-32 and CCC-39 with the treatment of bafilomycin A1 (*n* = 30). (D) The filamentous cells percentage of *C. albicans* WT, CCC-32 and CCC-39 with the treatment of bafilomycin A1 (*n* = 5). (E) The effect of bafilomycin A1 on the relative expression of genes in cAMP-PKA pathway (*n* = 3). (F) The effect of bafilomycin A1 on the adhesion percent of *C. albicans* WT on HOK cells (*n* = 3). (G) The images of *C. albicans* WT adhere to HOK cells. (H) The images of fluorescence staining of *C. albicans* hyphae invade into HOK cells. Cell wall chitin is stained with Calcofluor White (CFW, post-permeabilization), whereas carbohydrates are shown by Alexa-Fluor-647-labeled concanavalin A (ConA, pre-permeabilization), which respectively distinguish between invading hyphae and non-invading hyphae, and white arrows represent invading hyphae into HOK cells. (I) *C. albicans* burdens obtained from the tongues of mice after oropharyngeal infection (*n* = 6). (J) The images of infected mice tongues, leukoplakia on tongue are labelled by dotted lines. (K) HE staining of tongue tissues of mice, inflammatory cells and infiltrating immune cells are exhibited in black boxes. (L) PAS staining of tongue tissues of mice, invading hyphae are exhibited in black boxes. (ns no significant, * *p* < 0.05, ** *p* < 0.01, *** *p* < 0.001).

## Discussion

In this study, we characterized *C. albicans* V-ATPase V_0_c' subunit contributions to pathogenicity in oral epithelial infection. Importantly, the V_0_c' subunit encoding gene loss downregulated *C. albicans* proliferation and stress response and further weakened its virulence in inducing oral candidiasis *in vitro* and *in vivo*. Additionally, targeting the V-ATPase c ring effectively suppressed *C. albicans* hyphal development and epithelial pathogenesis (Figure 6). These results imply that the V_0_c' subunit is crucial for epithelial infection by *C. albicans* and V-ATPase may be a potential antifungal target against oral candidiasis.

**Figure 6.**
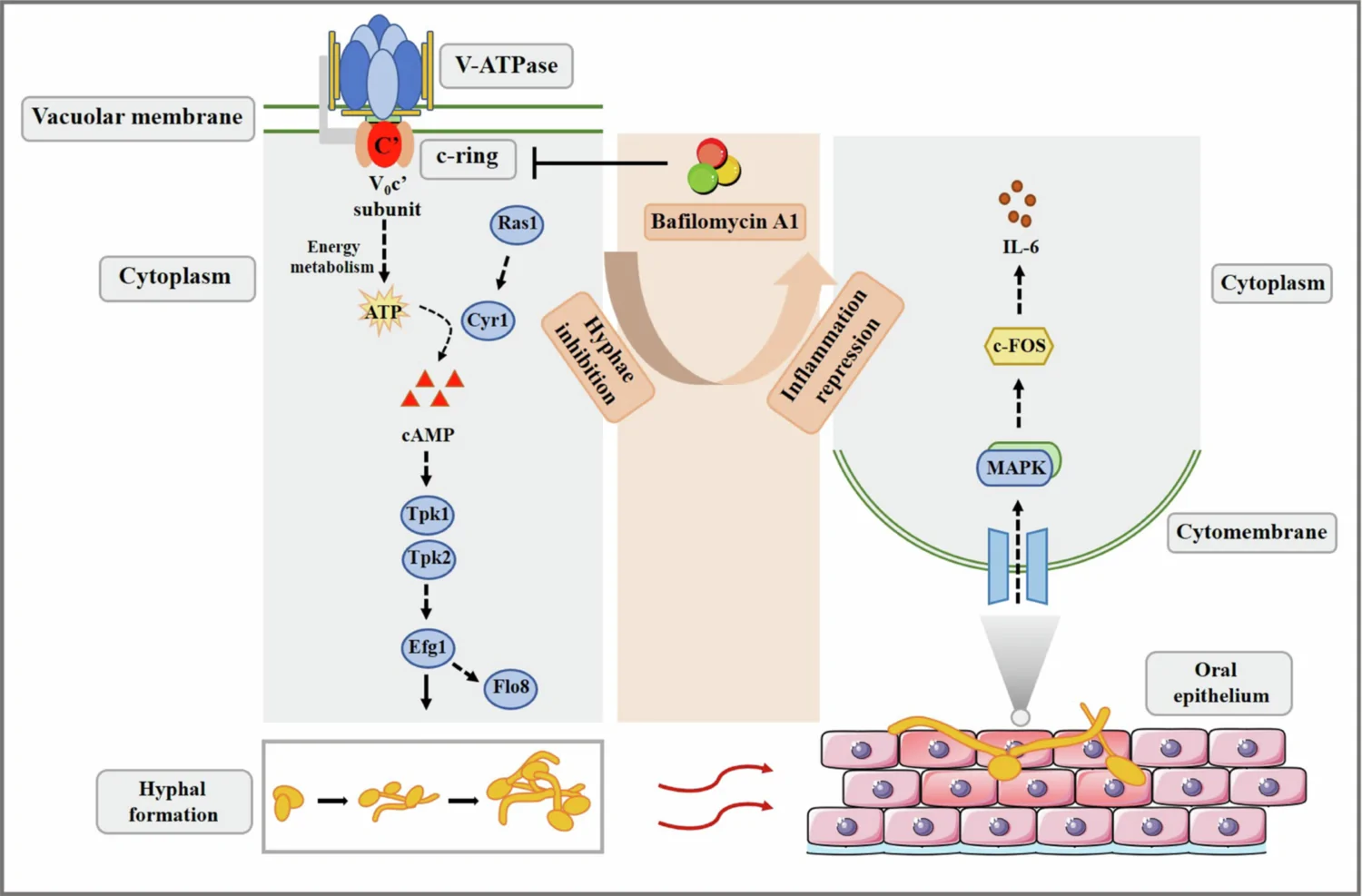
*C. albicans VMA11* mediated hyphal growth and oral epithelial invasion, and a V-ATPase c ring targeted strategy. During the epithelial infection process of *C. albicans*, *VMA11* regulates hyphal growth and influences the expression of cAMP-PKA pathway, in which *VMA11* modulates intracellular ATP synthesis and its downstream product cAMP, thereby affecting the expression of the transcription factor Efg1 to integrate multiple signals for hyphal morphogenesis. Then the pathogenic fungi break through the epithelial cell barrier, and induce MAPK and FOS-related signaling pathways involved in immune responses to mediate inflammatory factor production (such as IL-6). Targeting the V-ATPase c ring by specific inhibitor bafilomycin A1 exhibits significant hyphae inhibition ability and protects mice from oral candidiasis.

As an essential component of V_0_ domain, the c ring provides a channel that transports H^+^ into the vacuolar lumen. A previous study reported that V_0_c subunit repression displayed abnormal vacuolar morphology, defective pH-dependent growth and sensitivity to cold and calcium [23]. However, little is known about V_0_c' subunit function, as only one study reported that *VMA11* loss rendered *C. albicans* is unable to utilize haemoglobin as an iron source [39]. From our results, it was evident that the defective endocytosis function of the vacuole prevented *vma11Δ*/*Δ*-mediated H^+^ transport from the cytosol into the vacuolar lumen, thus making the mutant sensitive to acidic and alkaline medium, concomitant with a lack of compensation from other c ring genes. This observation was fundamentally different to previous findings where vacuolar pH dysfunction of V_0_a subunit mutant was not sufficient to impair pH-dependent growth and other virulence-related phenotypes. We hypothesize that the incomplete or incorrect assembly of any c ring subunit could eliminate V-ATPase function completely. The appearance of multiple membrane compartments within vacuole was another phenotype changing of *vma11Δ*/*Δ* compared to WT. Raymond et al. categorized mutant fungal vacuole morphology into six distinct phenotype classes [40]. In our study, we tend to characterize vacuolar morphology in *vma11Δ*/*Δ* mutant as class B, which is described as a ‘fragmented’ vacuole containing more than 20 subcompartments that cluster in the cytoplasm. It is currently accepted that vacuolar morphology is determined by the balance of two antagonistic and continuously occurring processes: fission and fusion. When the fusion process is more strongly affected than fission, this means that fission outweighs fusion, resulting in a cell with fragmented vacuoles [41]. Our results suggest that the fusion process may be disturbed upon deletion of *VMA11*.

We also showed that an absent *VMA11* significantly decreased *C. albicans* hyphal growth, which is regulated by complex signal transduction networks, with several major regulatory pathways identified in recent decades. Negative regulation is controlled by the transcription factor Tup1 in association with Nrg1 or the mycelial growth regulation factor Rfg1. Previous studies have confirmed that a lack of these negative regulation genes induce pseudohyphae formation and inhibit specific mycelium gene expression [42,43]. However, our data revealed that these repressors were not compensatorily activated in *vma11Δ*/*Δ*, suggesting that this pathway is not involved in V_0_c' subunit-mediated hyphal formation.

Positive regulators of hyphal growth include cAMP-PKA and MAPK pathways, which are generally mediated by the transcription factors Efg1 and Cph1, respectively. It is worth noting that MAPK pathway is not required for hyphal formation in liquid media since *CPH1* and upstream genes (*CST20*, *HST7* and *CEK1*) deletion failed to develop hypha on solid culture medium [44,45]. In the present study, although *VMA11* loss suppressed partial genes in MAPK pathway, *vma11Δ*/*Δ* did not form hyphae in both solid and liquid media conditions, and that there was no significant difference in the phospho-Cek1/total Cek1 ratio between *C. albicans* strains. Therefore, we hypothesize that the V_0_c' subunit may regulate hyphal morphogenesis by influencing the cAMP-PKA pathway. As expected, *VMA11* deletion significantly blocked the cAMP-PKA pathway and inhibited multiple virulent factors (*ALS1*, *ALS3* and *SSA1*). The second messenger cAMP is a key regulator in cAMP-PKA pathway activation. In responses to environmental stimulus such as serum, amino acids, and carbon dioxide, *C. albicans* expresses adenylyl cyclase (Cyr1) and catalyze precursor substrate ATP to produce cAMP [9]. Our results indicated decreased intracellular ATP and cAMP concentrations after *VMA11* deletion, while cAMP addition restored *vma11Δ*/*Δ* filamentation. As the downstream effector of cAMP, PKA is activated by the binding of its inhibitory subunit BCY1 and cAMP [46]. However, in our study, gene and protein expression data for PKA following *VMA11* inhibition were inconclusive, further research is required to characterize V_0_c' subunit effects on PKA activity and the phosphorylation status of downstream effector proteins.

Although our results revealed that the defect of hyphal formation caused by *VMA11* deletion involves significant changes in cAMP-PKA pathway, it remains inconclusive whether *VMA11* directly regulates hyphal development through this pathway. Previous studies have reported that inhibition of another c ring subunit-encoding gene *VMA3*, also suppresses hyphal formation. And the filamentation defect cannot be rescued by overexpression of positive regulators such as *RIM8*, *MDS3*, *EFG1*, *CST20*, or *UME6*, suggesting that the V-ATPase may function downstream of these transcriptional regulators or may be involved in an independent mechanism of hyphal formation. Therefore, we hypothesize that *VMA11* is unlikely to function as a canonical signaling component that directly regulate the cAMP–PKA pathway. Instead, as part of the V-ATPase c ring, V_0_c' subunit may maintain the intracellular physiological conditions required for efficient signaling. Loss of *VMA11* is expected to impair vacuolar proton transport, intracellular pH homeostasis, and mitochondrial function, thereby affecting ion balance and cellular energy metabolism. This hypothesis has been observed in *VMA3* mutants, where the loss of *VMA3* leads to blocked protons transport into the vacuole and defective mitochondrial function, consequently disrupting multiple downstream essential cellular processes [23,47]. Therefore, the loss of *VMA3* reduce intracellular ATP levels and contribute to decreased cAMP production since ATP is the substrate for adenylyl cyclase. This may subsequently reduce PKA activity and compromise hyphal development. Nevertheless, these relationships remain inferential, and direct measurements of the Co-IP between Vma11 and Ras proteins, the overexpression of key transcription factors in *vma11Δ*/*Δ* and metabolic flux will be required to establish the precise mechanistic sequence.

During *C. albicans* invasion into epithelial cells, the host mounts a complex immune-related cellular response. Pattern recognition receptors (PRRs) on host cell surfaces recognize pathogen-associated molecular patterns (PAMPs) expressed by fungi, with the subsequent activation of MAPK1 and FOS-related pathways that release proinflammatory cytokines and immune cell chemokines (interleukin-1α (IL-1α), interleukin-1β (IL-1β), interleukin-8 (IL-8), interleukin-17 (IL-17), interleukin-22 (IL-22) and chemokine ligand 20 (CCL20)), to recruit more immune cells in the infected area and improve the immune response, such as macrophages and dendritic cells [48]. Macrophages can participate in antifungal effect through phagocytosis and the secretion of cytokines (IL-1α, IL-1β, IL-8, colony-stimulating factor, CCL20), and they can also induce neutrophils to produce reactive oxygen species to kill *C. albicans* cells, which cannot be engulfed. Dendritic cells also play an important role in resisting *C. albicans* infection by activating T helper (Th) cells through antigen presentation. Th17 activation induces IL-17 and IL-22 production, which contribute to the recruitment and activation of neutrophils. IL-17 and IL-22 can further activate epithelial cells and promote the release of *β*-defensins. From our results, *C. albicans* infection induced the activation of several pathways (MAPK, HIF-1, IL-17 and TNF signaling pathways), with differentially expressed genes enriched in the MAPK signaling pathway between the WT and *vma11Δ*/*Δ* groups, suggesting that *VMA11*-regulated oral epithelial cell impairment is involved in MAPK signaling, similar to recent findings that MAPK is a key pathway in oral epithelial cells immune responses [44,49]. Among the most common identified proinflammatory cytokines mediating oral epithelial cell responses to *C. albicans* infection, IL-8, IL-17 or IL-22 were not detected, while IL-1α and IL-1β genes expression showed no statistically significant differences between WT and *vma11Δ*/*Δ*. However, *VMA11* deletion decreased IL-6 transcription levels, suggesting an implication in responses against *C. albicans*. Gene and protein expression assays further confirmed that MAPK-mediated Fos and IL-6 secretion significantly changed when response to *VMA11* regulated epithelial infection. Our results agree with previous studies that used multiple cell types, including oral, liver sinusoidal and vaginal epithelial cells, which showed that *C. albicans* infection activated MAPK signaling and promoted IL-6 release [44].

These results still have certain limitations. The response of the oral epithelium to *C. albicans* involves a complex network, and in the present study, IL-6 was selected as a representative marker of epithelial inflammation, which should not be interpreted as a comprehensive measure of epithelial antifungal immunity. For example, IL-6 is one of the earliest cytokines produced by epithelial cells in response to fungal infection and can be rapidly induced early during infection [50], consistent with the 6 h pre-treatment in our transcriptome sequencing analysis. In contrast, IL-17 depends on the activation of Th17 cells rather than direct secretion by epithelial cells [51]. Therefore, the lack of significant enrichment of IL-17 in epithelial transcriptome analysis is expected. Furthermore, epithelial cells from different mucosal sites may exhibit markedly distinct response profiles to *C. albicans* [52,53]. We hypothesize that the epithelial cell type used in the present study may have an intrinsic immune response profile more inclined toward IL-6 activation, with relatively weaker responses via IL-8 or IL-17. Thus, future studies should consider expanding the selection of epithelial cell lines and incorporating multiple infection time points as improvements.

Although *vma11Δ*/*Δ* exhibited significantly impaired hyphal formation, its attenuated pathogenicity against epithelial cells should not be attributed exclusively to disruption of a single phenotype or signaling pathway. Factors such as impaired growth capacity, and weakened stress adaptation to acid-base, metal, and oxygen stimuli may collectively contribute to the reductions of in vivo fungal burden and tissue damage. Although an equivalent initial inoculum was used in the infection assays, differences in fungal proliferation or survival during infection cannot be fully excluded.

Recent cryo-EM structural studies and the sequence alignments show that bafilomycin A1 cannot trigger the large-scale conformational changes of V-ATPase, as the binding residues of bafilomycin A1 are highly conserved among yeast and mammals. Bafilomycin A1 binds exclusively to the inter-subunit site formed by two adjacent c subunits, disrupting the interaction between the c ring and the V_0_a subunit, thereby blocking proton translocation. Residues M53, I56, V60, and I67 of one c subunit exhibit hydrophobic interaction with bafilomycin A1, while another c subunit forms ligand contacts via L133, F137, V140, and Y144 [38]. Therefore, bafilomycin A1 is a poor therapeutic candidate because it cannot discriminate between mammalian and fungal V-ATPases, posing potential host toxicity, especially as we found that the MIC of bafilomycin A1 on WT was same to *vma11Δ*/*Δ* (Table S2). For the present study, bafilomycin A1 should be regarded as a pharmacological proof-of-concept tool demonstrating that interfering with V-ATPase activity affects the filamentation and oral epithelial pathogenicity of *C. albicans*. In mammals, the c and c'' subunits form the c ring, whereas in *C. albicans*, the c ring is organized into c, c', and c'' subunits [54,55]. The V_0_c' subunit lacks a homolog in mammals, further supporting its potential as a drug target. Our future work will focus on elucidating the molecular basis of the V_0_c' subunit and rationally designing potential drugs that specifically target it.

## Conclusion

In conclusion, our study highlights V-ATPase V_0_c' subunit contributions to *C. albicans* pathogenicity in oral epithelial infection. By initially revealing the mechanisms of *VMA11* regulated hyphal development and oral epithelial cells damage, we provide novel insights into antifungal targets, with the rising concern about antifungal resistance. Future work focusing on the development of V_0_c' subunit precisely targeted drugs.

## Supplementary Material

Supporting_Materials (1).docx

## Data Availability

The transcriptome sequencing data have been deposited in the NCBI database (Accession numbers: PRJNA1273333). All other data that support the findings of this study are available from the corresponding author upon reasonable request.
