# Correction

**DOI:** 10.1080/20002297.2026.2760660

**Published:** 2026-11-08

**Article title:** Correction to: The V-ATPase V0c' subunit is critical for Candida albicans pathogenicity in oral epithelial infection.

**Authors:** Zheng Wang, Shenglan Long, Han Du, Xiaomeng Liu, Yiding Li, Qian Jiang, Lang Cai, Jing Zhou, Deqin Yang

**Journal:** *Journal of Oral Microbiology*

**Bibliometrics:** Volume 18, Issue 1, Pages 1−21

**DOI:** 10.1080/20002297.2026.2739029

Correction Notice:

In the originally published version of this article, an inadvertent error was made during the assembly of the figures. Specifically, the control group image for Figure 5G was mistakenly swapped with another image. The corrected version of Figure 5G has been updated in the article.

