# Supplementary material for: The V-ATPase V 0 c' subunit is critical for Candida albicans pathogenicity in oral epithelial infection
Source: J Oral Microbiol. 2026 Sep 30;18(1):2739029. doi: 10.1080/20002297.2026.2739029 (PMC13629789; doi:10.1080/20002297.2026.2739029)
Supplement: Supplementary file 1 [file ZJOM_A_2739029_SM7864.docx]

**Construction of *VMA11* complementation strain**

To generate the *VMA11* reconstituted plasmid, fragments of the 3′- untranslated region (UTR) and those containing the open reading frame (ORF) plus 5′-UTR of *VMA11* were amplified by polymerase chain reaction (PCR) from SN250 genomic DNA. PCR products were digested with Sac II/Sac I and Apa I/Xho I, respectively, and subcloned into the pSFS2A plasmid. Then the pSFS2A-VMA11p-VMA11 plasmid was digested with ApaI/SacI and transformed into *vma11Δ*/*Δ* mutant to construct *VMA11* complementation strain.

**Growth curve assay**

Briefly, overnight *C. albicans* cells were harvested and sub-cultured in fresh YPD medium, grown until mid-exponential phase, and resuspended in YPD medium (1×10^5^ cells/ml). After covering with sterile mineral oil, cultures were incubated at 35 °C. The optical density at 600 nm was recorded at 1 h interval during the 24 h growth period using a spectrophotometer (microquant microplate spectrophotometer; BioTek Instruments Inc, Winooski, Vt., U.S.A.).

**The colony morphology** **observation**

For colony morphology, fungal suspension was serially diluted in PBS, plated onto agar containing the above medium, and incubated at 37℃ for 48 h. A stereomicroscope (Leica, Germany) was used to observe the colonies.

**The effects of exogenous cAMP and bafilomycin A1 on hypha induction**

For the exogenous cAMP treatment, mid-logarithmic phase cells were resuspended in fresh YPD medium containing cAMP (0 μM, 5μM and 10μM) and cultured at 37 °C for 24 h, after which hyphae were visualized using an inverted microscope. To examine the influence of fluconazole and bafilomycin A1 on hypha formation, *C. albicans* cells at mid-logarithmic phase were resuspended in fresh YPD medium (1×10^5^ cells/ml) and suspensions added to a 96-well plate. Dimethyl sulfoxide (DMSO) was used as the negative control, and fluconazole (5 μg/mL) and bafilomycin A1 (0.1 and 0.5 μM) were added to test wells. Plates were incubated at 37 °C for 24 h and hyphae visualized by microscopy (Leica, Germany).

**Scanning electron microscopy** **hyphal morphology observation**

*C. albicans* in logarithmic growth phase (YPD medium contained 10% serum) was washed three times with PBS, centrifuged at 4000 g for 10 minutes to discard the supernatant, fixed overnight in 1 ml of 4% glutaraldehyde at 4°C, and serial dehydrated with ethanol (50%, 60%, 70%, 80%, 90%, 95%, and 100%). Samples were then coated with gold for SEM imaging (FEI, Hillsboro, USA).

**Analysis of Cek1 phosphorylation.**

For Cek1 phosphorylation studies, mid-exponential phase *C. albicans* cells were resuspended in spider medium containing 0.2% glucose (1×10^5^ cells/ mL) and cultured at 37 °C for 12 h. Then *C. albicans* cells were washed with PBS and resuspended in radio immunoprecipitation assay (RIPA) lysis buffer (Beyotime, China) containing 1mM protease inhibitor cocktail (Beyotime, China) and 1mM phosphatase inhibitor (Beyotime, China). Total protein was extracted using a FastPrep-24 homogenizer (MP Biomedicals, Santa Ana, CA, USA). Proteins were quantified by bicinchoninic acid (BCA) assay kit (Beyotime, China). All protein samples were then boiled for 10 min at 100 ℃. Equal amounts of proteins (25 μg in total) were separated by 10 % (w/v) sodium dodecyl sulfate-polyacrylamide gel electrophoresis (SDS-PAGE) (Epizyme, USA) and transferred to Immobilon®-FL transfer membranes (Millipore, USA). Membranes were blocked with 5% nonfat milk or 5% BSA for 2 h and incubated separately with 1:1000 diluted primary antibodies (Yeast β-actin, RRID: YM_8343; p-Cek1, RRID: R_24245; Cek1, RRID: 343830) at 4 ℃ overnight, respectively. After that, membranes were incubated with HRP-tagged goat anti-rabbit IgG (1:3000, CST) for 2 h at room temperature. Membranes were then visualized using a BeyoECL Plus kit (Beyotime, China), followed by protein bands quantification using Image J software.

**PKA activity and intracellular ATP and cAMP content assays**

For PKA activity assay, the absorbances at 340 nm of total protein were measured at initial time and fifth minute following the PKA Colorimetric Activity Kit manufacturer’s instructions (GenMed). The difference in readings between two time points represents the PKA activity. For ATP and cAMP content assays, mid-exponential phase *C. albicans* cells were resuspended in fresh YPD medium (1×10^5^ cells/ mL) and cultured at 37 °C for 4 h. Then, 100 ul of the suspended cells were diluted by PBS for CFU counts. *C. albicans* cells were collected and washed with PBS. Samples were then evenly divided in two, with one used for ATP assay and the other for cAMP detection. For ATP measurements, *C. albicans* cells were resuspended in 200 μL PBS in grinding tubes with glass bead and broken in FastPrep-24™ 5G bead beating grinder (MP Biomedicals, CA, USA). For cAMP measurement, *C. albicans* cells were resuspended in 200 μL 0.1M HCL and broke broken in a bead beating grinder as described. All samples were subsequently centrifuged at 12000 g for 10 minutes at 4°C. ATP and cAMP concentration in supernatants were measured using ATP Assay Kit (Beyotime, China) and cAMP enzyme-linked immunosorbent assay (Elisa) Kit (ewEAST Biosciences, China) according to manufacturer's instructions, respectively. Relative ATP and cAMP levels were expressed as concentration to CFU counts ratios.

**Epithelial cell adhesion and invasion assays**

For epithelial cell adhesion assay, HOK cells were inoculated into confocal dishes and cultured for 24 h (37 °C, 5% CO_2_, humid environment). Then HOK cells were co-culture with *C. albicans* strains (1×10^5^ cells/ml) for 1 h. After removing the medium, it was washed three times with PBS to thoroughly remove unattached *C. albicans* cells. Samples were divided into two; one was incubated with 1 ml sterile double-distilled water at 37 °C for 1 h to lyse HOK cells, with suspensions gradually diluted and added to YPD plates to determine CFU counts. The other sample was fixed with 4% paraformaldehyde for gram staining according to the instructions. Three fields of view were randomly selected for observations and image acquisition. To assess the impact of bafilomycin A1 on *C. albicans* adhesion, HOK cells were co-culture with *C. albicans* suspension including bafilomycin A1 at a final concentration of 0.1 μM and 0.5 μM for 1 h. DMSO was used as negative control. After removing the medium and washing cells with PBS, samples were fixed and visualized.

For epithelial cell invasion assay, HOK cells were inoculated into confocal dishes and cultured for 24 h (37 °C, 5% CO_2_, humid environment). HOK cells were then co-culture with *C. albicans* strains (1×10^5^ cells/ml) for 3 h. The medium was removed and washed three times with PBS, after which samples were fixed overnight in 4% paraformaldehyde. Samples were stained with Concanavalin A-Alexa Fluor 647 (10 µg/ml, Beyotime, China) at room temperature in the dark for 45 min (The hyphae that invaded the cells were not stained with Concanavalin A) and washed three times with PBS. HOK cells were then infiltrated with 0.1% Triton X-100 for 15 min and stained with CFW (Beyotime, China) for 20 min in the dark (Both intracellular and extracellular hyphae were stained with CFW). After washing with PBS, confocal dish was then visualized under an inverted fluorescence microscope (TCS. SP8, Leica, Germany). In the overlay field of view, the areas that were stained with blue but not red represented the hyphae that invaded the cells. Three fields of view of each sample were randomly observed for image collection. To assess the impact of bafilomycin A1 on *C. albicans* invasion, HOK cells were co-cultured with *C. albicans* suspension including bafilomycin A1 at a final concentration of 0.1 μM and 0.5 μM for 3 h. DMSO was used as negative control. After removing the medium and washing with PBS, samples were fixed, stained and visualized.

**RNA-Sequencing**

Total RNA was extracted from the tissue using TRIzol® Reagent according the manufacturer’ s instructions. Then RNA quality was determined by 5300 Bioanalyser (Agilent) and quantified using the ND- 2 0 0 0 (NanoDrop Technologies). Only high-quality RNA sample (OD260/280= 1.8~2.2, OD260/230≥2.0, RQN≥ 6.5, 28S:18S≥ 1.0, >1g) was used to construct sequencing library.

RNA purification, reverse transcription, library construction and sequencing were performed at Shanghai Majorbio Bio-pharm Biotechnology Co., Ltd. (Shanghai, China) according to the manufacturer’s instructions (Illumina, San Diego, CA). The RNA-seq transcriptome librariy was prepared following Illumina® Stranded mRNA Prep Ligation from Illumina (San Diego, CA) using 1μg of total RNA. Shortly, messenger RNA was isolated according to polyA selection method by oligo(dT) beads and then fragmented by fragmentation buffer firstly. Secondly doublestranded cDNA was synthesized using a SuperScript double-stranded cDNA synthesis kit (Invitrogen, CA) with random hexamer primers (Illumina). Then the synthesized cDNA was subjected to end-repair, phosphorylation and ‘A’ base addition according to Illumina’s library construction protocol. Libraries were size selected for cDNA target fragments of 300 bp on 2% Low Range Ultra Agarose followed by PCR amplified using Phusion DNA polymerase (NEB) for 15 PCR cycles. After quantified by Qubit 4.0, paired-end RNA-seq sequencing library was sequenced with the NovaSeq X plus sequencer (2 × 150bp read length).

The raw paired end reads were trimmed and quality controlled by fastp with default parameters. Then clean reads were separately aligned to reference genome with orientation mode using HISAT2 software. The mapped reads of each sample were assembled by StringTie in a reference- based approach.

To identify DEGs (differential expression genes) between two different samples, the expression level of each transcript was calculated according to the transcripts per million reads (TPM) method. RSEM was used to quantify gene abundances. Essentially, differential expression analysis was performed using the DESeq2 or DEGseq. DEGs with |log2FC| ≧1 and FDR≤ 0.05(DESeq2) or FDR ≤0.001(DEGseq) were significantly different expressed genes. In addition, functional- enrichment analysis including GO and KEGG were performed to identify which DEGs were significantly enriched in GO terms and metabolic pathways at Bonferroni-corrected P-value ≤0.05 compared with the whole-transcriptome background. GO functional enrichment and KEGG pathway analysis were carried out by Goatools and KOBAS, respectively.

All the alternative splice events that occurred in our sample were identified by using recently releases program rMATS. Only the isoforms that were similar to the reference or comprised novel splice junctions were considered, and the splicing differences were detected as exon inclusion, exclusion, alternative 5′, 3′, and intron retention events.

**RT-qPCR detection and western blot analysis of HOK cells**

As described in transcriptome sequencing protocol, HOK cells were co-cultured with *C. albicans* for 6 h. For RT-qPCR, HOK cells were washed three times with PBS and lysed by Trizol lysis buffer, with a subsequent RNA extraction using TRIzol Reagent (Invitrogen Life Technologies, USA) according to manufacturer’s instructions. RNA samples were purified and reverse transcribed using a PrimeScript^TM^ RT reagent Kit (Takara, Japan). Real-time PCR was performed by a Bio-Rad CFX96 real-time PCR detection system with SYBR Green qPCR mix (TOYOBO, Japan). All data were analyzed using the 2^-ΔΔCT^ method and compared to *GAPDH* rRNA levels.

For western blot analysis, HOK cells were washed three times with PBS and lysed using RIPA lysis buffer (Beyotime, China) containing 1mM protease inhibitor cocktail (Beyotime, China). Proteins were quantified by BCA assay kit (Beyotime, China). All protein samples were then boiled for 10 min at 100 ℃. Equal amounts of proteins (25 μg in total) were separated by 10 % (w/v) SDS-PAGE (Epizyme, USA) and transferred to Immobilon®-FL transfer membranes (Millipore, USA). Membranes were blocked with 5% nonfat milk or 5% BSA for 2 h and incubated separately with 1:1000 diluted primary antibodies (β-actin, RRID: AB_330288; c-FOS, RRID: AB_222699; IL-6, RRID: AB_6672) at 4 ℃ overnight, respectively. After that, membranes were incubated with HRP-tagged goat anti-rabbit IgG (1:3000, CST) for 2 h at room temperature. Membranes were then visualized using a BeyoECL Plus kit (Beyotime, China), followed by protein bands quantification using Image J software.

**Cytotoxicity test of** **Bafilomycin A1**

In brief, 5000 HOK cells/well suspended in 100 μL DMEM culture medium (Gibco, USA) was used in 96-well plates and incubated with 0.1 μL bafilomycin A1 (0 uM, 0.065 uM, 0.125 uM, 0.25 uM, 0.5 uM and 1 uM) for 24 h at 37℃ with 5% CO_2_. Then, the DMEM culture medium (Gibco, USA) was discarded and replaced with 100 uL of fresh culture medium and 10 uL of etrazolium-8-[2-(2-methoxy-4-nitrophenyl)-3-(4-nitrophenyl)-5-(2,4-disulfophenyl)-2H-tetrazolium] monosodium salt (CCK-8 solution). After incubating for 1 hour, the solution absorbance was measured at 450nm by a Thermo Scientific Multiskan GO reader (Thermo Fisher Scientific Inc., Waltham, MA, USA).

**Figure S1** (**A**) The growth kinetics of *C. albicans* strains during the 20-h cell growth period (*n* = 6). (**B**) Growth of *C. albicans* strains under acidic and alkaline stress.

**Figure S2** (**A**) The colony morphology of *C. albicans* strains in YPD and Spider solid media. (**B**) The SEM images of hyphae formation of *C. albicans* strains under 2000 × and 3000 × magnified visual field. (**C**) The relative expression of genes in Tup1 mediated negative regulation pathway (n = 3). (**D**) The relative expression of genes in MAPK pathway (n = 3). (ns no significant, ***** *p* < 0.05, ****** *p* < 0.01, ******* *p* < 0.001)

**Figure S3** (**A**) KEGG enrichment analysis between WT and cell groups. (**B**) KEGG enrichment analysis between *vma11Δ*/*Δ* and cell groups.

**Table S1 The top60 downregulated genes in *vma11Δ*/*Δ* infected HOK cells compared to wild type infected HOK cells of transcriptome sequencing (Differential expression genes with |log_2_FC| ≧1 and FDR≤ 0.05(DESeq2) or FDR ≤0.001(DEGseq) were significant)**

| **Gene id** | **Gene name** | **FC** | **Log_2_FC** | **Pvalue** | **Padjust** | **Significant** |
| --- | --- | --- | --- | --- | --- | --- |
| ENSG00000288725 | - | 0.082051 | -3.60733 | 1.78E-05 | 0.000349 | yes |
| ENSG00000170236 | USP50 | 0.114653 | -3.12465 | 4.48E-05 | 0.000783 | yes |
| ENSG00000268790 | - | 0.116949 | -3.09605 | 6.04E-05 | 0.001013 | yes |
| ENSG00000125740 | FOSB | 0.122823 | -3.02535 | 0 | 0 | yes |
| ENSG00000173110 | **HSPA6** | 0.198741 | -2.33104 | 6.58E-21 | 9.96E-19 | yes |
| ENSG00000188766 | SPRED3 | 0.208491 | -2.26195 | 0.00033 | 0.004332 | yes |
| ENSG00000100292 | HMOX1 | 0.210052 | -2.25118 | 4.27E-160 | 1.55E-156 | yes |
| ENSG00000170345 | **FOS** | 0.216371 | -2.20842 | 0 | 0 | yes |
| ENSG00000246214 | RETREG1-AS1 | 0.219146 | -2.19004 | 0.000854 | 0.009615 | yes |
| ENSG00000289523 | - | 0.2363 | -2.08131 | 0.002213 | 0.020422 | yes |
| ENSG00000263624 | - | 0.258525 | -1.95162 | 0.000313 | 0.004162 | yes |
| ENSG00000225434 | LINC01504 | 0.269476 | -1.89177 | 6.79E-06 | 0.000151 | yes |
| ENSG00000273149 | - | 0.274167 | -1.86687 | 0.004086 | 0.033666 | yes |
| ENSG00000249007 | - | 0.281161 | -1.83053 | 0.002658 | 0.023551 | yes |
| ENSG00000006606 | CCL26 | 0.282214 | -1.82514 | 1.14E-09 | 5.61E-08 | yes |
| ENSG00000162772 | ATF3 | 0.302446 | -1.72525 | 2.96E-229 | 1.43E-225 | yes |
| ENSG00000177483 | RBM44 | 0.303559 | -1.71995 | 0.002558 | 0.022862 | yes |
| ENSG00000112309 | B3GAT2 | 0.30736 | -1.702 | 1.33E-05 | 0.000272 | yes |
| ENSG00000123358 | **NR4A1** | 0.310679 | -1.6865 | 1.83E-46 | 1.11E-43 | yes |
| ENSG00000119508 | NR4A3 | 0.317472 | -1.6553 | 8.25E-17 | 9.30E-15 | yes |
| ENSG00000245156 | - | 0.322748 | -1.63152 | 0.001496 | 0.014959 | yes |
| ENSG00000260633 | - | 0.329872 | -1.60002 | 0.00072 | 0.008286 | yes |
| ENSG00000119630 | **PGF** | 0.342266 | -1.54681 | 0.001137 | 0.012096 | yes |
| ENSG00000286360 | - | 0.362128 | -1.46543 | 0.00128 | 0.013227 | yes |
| ENSG00000227676 | LINC01068 | 0.369945 | -1.43462 | 4.44E-05 | 0.000779 | yes |
| ENSG00000232656 | IDI2-AS1 | 0.373281 | -1.42167 | 4.02E-08 | 1.42E-06 | yes |
| ENSG00000181322 | NME9 | 0.375069 | -1.41477 | 0.005079 | 0.039988 | yes |
| ENSG00000272037 | - | 0.387682 | -1.36705 | 0.003831 | 0.031927 | yes |
| ENSG00000204388 | **HSPA1B** | 0.391868 | -1.35156 | 2.25E-23 | 3.99E-21 | yes |
| ENSG00000204389 | **HSPA1A** | 0.399856 | -1.32245 | 1.24E-16 | 1.35E-14 | yes |
| ENSG00000179148 | ALOXE3 | 0.402329 | -1.31355 | 0.003689 | 0.030902 | yes |
| ENSG00000279432 | - | 0.409079 | -1.28955 | 0.001574 | 0.015517 | yes |
| ENSG00000233885 | YEATS2-AS1 | 0.411273 | -1.28183 | 0.0001 | 0.001577 | yes |
| ENSG00000115844 | DLX2 | 0.4119 | -1.27963 | 0.006043 | 0.045797 | yes |
| ENSG00000185565 | LSAMP | 0.412798 | -1.27649 | 0.004515 | 0.036334 | yes |
| ENSG00000119986 | AVPI1 | 0.419372 | -1.2537 | 1.21E-47 | 8.36E-45 | yes |
| ENSG00000119938 | PPP1R3C | 0.421943 | -1.24488 | 3.34E-05 | 0.000608 | yes |
| ENSG00000214922 | HLA-F-AS1 | 0.423565 | -1.23934 | 0.002233 | 0.020566 | yes |
| ENSG00000144655 | CSRNP1 | 0.434746 | -1.20176 | 1.70E-36 | 6.69E-34 | yes |
| ENSG00000132002 | DNAJB1 | 0.435201 | -1.20024 | 3.51E-19 | 4.86E-17 | yes |
| ENSG00000261578 | - | 0.436448 | -1.19612 | 6.68E-05 | 0.001102 | yes |
| ENSG00000186480 | INSIG1 | 0.444619 | -1.16936 | 3.38E-95 | 5.47E-92 | yes |
| ENSG00000140403 | DNAJA4 | 0.447443 | -1.16023 | 5.82E-39 | 2.73E-36 | yes |
| ENSG00000087074 | PPP1R15A | 0.450573 | -1.15017 | 3.15E-85 | 4.58E-82 | yes |
| ENSG00000184557 | SOCS3 | 0.456061 | -1.1327 | 3.27E-09 | 1.45E-07 | yes |
| ENSG00000224431 | - | 0.456802 | -1.13036 | 0.001568 | 0.015489 | yes |
| ENSG00000153234 | NR4A2 | 0.463549 | -1.10921 | 2.65E-15 | 2.59E-13 | yes |
| ENSG00000186352 | ANKRD37 | 0.466215 | -1.10093 | 1.20E-16 | 1.31E-14 | yes |
| ENSG00000286223 | - | 0.469921 | -1.08951 | 0.003843 | 0.03199 | yes |
| ENSG00000178381 | ZFAND2A | 0.470871 | -1.0866 | 5.04E-25 | 1.02E-22 | yes |
| ENSG00000153714 | LURAP1L | 0.471667 | -1.08416 | 3.11E-05 | 0.000573 | yes |
| ENSG00000142178 | SIK1 | 0.475221 | -1.07333 | 5.75E-58 | 4.92E-55 | yes |
| ENSG00000164400 | CSF2 | 0.477104 | -1.06763 | 1.59E-06 | 4.07E-05 | yes |
| ENSG00000179388 | EGR3 | 0.479906 | -1.05918 | 3.29E-06 | 7.87E-05 | yes |
| ENSG00000291266 | SMG1P5 | 0.485501 | -1.04245 | 1.22E-07 | 3.99E-06 | yes |
| ENSG00000120129 | **DUSP1** | 0.486878 | -1.03837 | 6.12E-29 | 1.65E-26 | yes |
| ENSG00000204531 | POU5F1 | 0.489005 | -1.03208 | 2.16E-05 | 0.000413 | yes |
| ENSG00000280138 | - | 0.490028 | -1.02906 | 1.47E-11 | 9.27E-10 | yes |
| ENSG00000283761 | - | 0.494595 | -1.01568 | 0.000358 | 0.004659 | yes |

**Figure S4** (**A**) The effect of bafilomycin A1 on the relative expression of *VMA11* (n = 3). (**B**) Hyphae formation of *C. albicans* WT, CCC-32 and CCC-39 with the treatment of fluconazole in YPD medium. (**C**) The hyphal length of *C. albicans* WT, CCC-32 and CCC-39 with the treatment of fluconazole (n = 30). (**D**) The filamentous cells percentage of *C. albicans* WT, CCC-32 and CCC-39 with the treatment of fluconazole (n = 5). (**E**) Cytotoxicity assay of bafilomycin A1 with HOK cells (n=3). (**F**) HE-stained sections of major organs (Liver, kidney and spleen) from mice subjected to topical application of bafilomycin A1. (ns no significant, ******* *p* < 0.001)

**Table S2 The inhibitory effects of bafilomycin A1 against *C. albicans* wild type, *vma11Δ*/*Δ* and *vma11Δ*/*Δ*+*VMA11***

| **Strain** | **MIC** |
| --- | --- |
| Wild type (SN250) | 0.5 μM |
| *vma11Δ*/*Δ* | 0.5 μM |
| *vma11Δ*/*Δ*+*VMA11* | 0.5 μM |
